# Relationships between paraspinal muscle morphology and neurocompressive conditions of the lumbar spine: a systematic review with meta-analysis

**DOI:** 10.1186/s12891-018-2266-5

**Published:** 2018-09-27

**Authors:** Jeffrey R. Cooley, Bruce F. Walker, Emad M. Ardakani, Per Kjaer, Tue S. Jensen, Jeffrey J. Hebert

**Affiliations:** 1 School of Health Professions, Murdoch University, 90 South Street, Murdoch, Western Australia 6150 Australia; 2 Department of Sports Science and Clinical Biomechanics, University of Southern Denmark, Campusvej 55, 5230, Odense M, DK Denmark; 3 Spine Centre of Southern Denmark, Ostre Hougvej 55, 5500 Middelfart, DK Denmark; 4 Nordic Institute of Chiropractic and Clinical Biomechanics, Campusvej 55, 5230 Odense M, DK Denmark; 5 Department of Diagnostic Imaging, Regional Hospital Silkeborg, Falkevej 1-3, 8600 Silkeborg, DK Denmark; 6 Faculty of Kinesiology, University of New Brunswick, 3 Bailey Drive, Fredericton, New Brunswick E3B 5A3 Canada; 7 School of Psychology and Exercise Science, Murdoch University, 90 South Street, Murdoch, Western Australia 6150 Australia

**Keywords:** Lumbar spine, paraspinal muscle, disc herniation, radiculopathy, fat infiltration, canal stenosis, facet arthrosis

## Abstract

**Background:**

Individual study results have demonstrated unclear relationships between neurocompressive disorders and paraspinal muscle morphology. This systematic review aimed to synthesize current evidence regarding the relationship lumbar neurocompressive disorders may have with lumbar paraspinal muscle morphology.

**Methods:**

Searches were conducted in seven databases from inception through October 2017. Observational studies with control or comparison groups comparing herniations, facet degeneration, or canal stenosis to changes in imaging or biopsy-identified lumbar paraspinal muscle morphology were included. Data extraction and risk of bias assessment were performed by review author pairs independent of one another. Morphological differences between individuals with and without neurocompressive disorders were compared qualitatively, and where possible, standardised mean differences were obtained.

**Results:**

Twenty-eight studies were included. Lumbar multifidus fiber diameter was smaller on the side of and below herniation for type I [SMD: −0.40 (95% CI = −0.70, −0.09) and type II fibers [SMD: −0.38 (95% CI = −0.69, −0.06)] compared to the unaffected side. The distribution of type I fibers was greater on the herniation side [SMD: 0.43 (95% CI = 0.03, 0.82)]. Qualitatively, two studies assessing small angular fiber frequency and fiber type groupings demonstrated increases in these parameters below the herniation level. For diagnostic imaging meta-analyses, there were no consistent differences across the various assessment types for any paraspinal muscle groups when patients with herniation served as their own control. However, qualitative synthesis of between-group comparisons reported greater multifidus and erector spinae muscle atrophy or fat infiltration among patients with disc herniation and radiculopathy in four of six studies, and increased fatty infiltration in paraspinal muscles with higher grades of facet joint degeneration in four of five studies. Conflicting outcomes and variations in study methodology precluded a clear conclusion for canal stenosis.

**Conclusions:**

Based on mixed levels of risk of bias data, in patients with chronic radiculopathy, disc herniation and severe facet degeneration were associated with altered paraspinal muscle morphology at or below the pathology level. As the variability of study quality and heterogeneous approaches utilized to assess muscle morphology challenged comparison across studies, we provide recommendations to promote uniform measurement techniques for future studies.

**Trial registration:**

PROSPERO 2015: CRD42015012985

**Electronic supplementary material:**

The online version of this article (10.1186/s12891-018-2266-5) contains supplementary material, which is available to authorized users.

## Background

Globally, low-back pain (LBP) ranks first in years lived with disability [1]. The lifetime prevalence of LBP is estimated to be as high as 84% [2], with a mean of 38.9% [3]. In Australia, 2001 estimates revealed a direct and indirect cost of LBP of AUD$9.17 billion [4]. In 2014, the estimated annual cost of chronic LBP-related lost productivity in Japan was ¥1.2 trillion (equivalent to AUD$12.6 billion) [5]. It should be noted that these estimated prevalence rates and costs are inclusive of all types of LBP; however, approximately 90% of LBP is non-specific in nature, while specific LBP resulting from an identifiable disorder (e.g., tumor, fracture, stenosis) can only be classified in a small percentage of patients [6]. Although there is very limited data available to quantify the prevalence of neuro-compressive disorders such as lumbar disc herniation, facet joint hypertrophy and lumbar spinal stenosis, these can only make up a portion of the 10% of specific LBP cases.

Despite intensive research efforts aimed at enhancing our understanding of both specific and non-specific LBP, these disorders continues to present diagnostic and therapeutic challenges. In an attempt to identify discrete pain generating tissues or clinically relevant structural changes related to LBP, recent studies have focused on the relationships between morphological changes to the lumbar paraspinal musculature (e.g., atrophy, fat replacement) and both specific and non-specific causes of chronic low back or radicular pain [7–11]. Systematic reviews have assessed the relationship of paraspinal muscle morphology with LBP, the impact of paraspinal muscle atrophy and/or fatty replacement on clinical outcomes, and the predictive value of paraspinal muscle morphology with clinical outcomes [12–14].

Of particular interest to this review is the growing body of research attempting to identify the relationships between spinal pathologies and paraspinal muscle morphology, and their impact on specific LBP and clinical outcomes [15–21]. One specific area of interest focuses on localized injuries or pathologies resulting in nerve root or central neurological compression (neurocompressive disorders), as it is understood that the biological effects of short and long-term skeletal muscle denervation can result in muscle fiber atrophy and adipose tissue replacement [22–24]. However, no prior systematic reviews of these relationships have been identified by the authors. A 2014 review by Steffens et al. [25], explored the ability of MRI-identified pathologies to predict future LBP, concluding that no definitive associations between imaging findings and clinical outcomes could be confirmed due to limited research in this area. However, these authors did not include altered muscle morphology in their pathology criteria, nor did they look at the relationship of paraspinal muscle morphology to regional pathology.

Therefore, the objective of this study was to systematically review the literature to investigate for relationships between lumbosacral neurocompressive disorders and measures of lumbar paraspinal muscle morphology in patients with specific LBP.

## Methods

### Protocol and registration

This review followed the reporting guidelines and methodologies proposed in *Preferred reporting items of systematic reviews and meta-analysis: the PRISMA statement* [26] and *Meta-analysis of observational studies in epidemiology* (MOOSE) [27]. The initial review protocol was registered with Prospero, 13 February 2015 (PROSPERO 2015:CRD42015012985), available from: http://www.crd.york.ac.uk/PROSPERO/display_record.asp?ID=CRD42015012985. The original search strategy was applied following the registered protocol; however, due to the large and diverse number of articles meeting the eligibility criteria, a post-hoc decision was made to the original protocol to limit this review to patients with radicular pain or reduced muscle strength in the lower extremities due to neurocompression.

### Information sources

With the assistance of specialist librarians, we developed a search strategy using medical subject headings (MeSH) and keywords that encompassed muscle type and morphology; pathology and related clinical syndromes; imaging types, biopsy analyses, and muscle measurement parameters; and, the lumbar spinal region. No language restrictions were applied.

We searched the following databases from inception through October 2017 in PEDro, PubMed (Medline), Web of Science (Core Collection), Web of Science (Medline Advanced), SPORTDiscus, Cumulative Index to Nursing and Allied Health Literature (CINAHL), and EMBASE. The reference lists of included studies from the title/abstract screening, as well as all systematic reviews related to the topic, were also reviewed. Where only an abstract was published as part of a poster or conference proceedings, the authors were contacted via email to determine if the full studies had since been published. The search protocols for each database can be found in Additional file 1.

### Eligibility and study selection criteria

The eligibility and selection criteria are provided in Table 1. The outcomes of interest included measures of lumbar paraspinal muscle morphology, such as muscle cross-sectional area, fat infiltration area, and type I and II muscle fiber distribution.

**Table 1:** Study eligibility and selection criteria

Inclusion criteria	
Articles published (including those accepted for publication) in an indexed, peer reviewed journal, or a published^a^ thesis Studies including patients with: disc herniation, facet arthrosis, and/or spinal canal stenosis identified via imaging; specific LBP with confirmed radicular leg pain or muscle weakness on clinical examination Regional paraspinal muscle morphology assessed with imaging or biopsy for either the lumbar multifidus muscles (LMM), erector spinae muscles (ESM) (including subcomponents), psoas major muscles (PMM), or "paraspinal / paravertebral" muscles (PVM) Observational human studies with a control or comparison analyses (controls included: "normal" or “non-diseased”; comparisons between different severities of conditions; participants serving as own control when there was a normal and an abnormal side to compare) Clinical / surgical trials containing baseline data with relevant “pathology to muscle” or “clinical to muscle” comparisons	
Exclusion criteria	
History of previous lumbar spine surgery Analysis was solely post-interventional (i.e., no pre-surgical, pre-treatment, or pre-activity/functional muscle measurement data analysed) Case reports, editorials/letters, literature reviews, guidelines, and abstract-only publications Patients with primary muscular disease (e.g., muscular dystrophy, parkinsonism)	

^a^If archived in an international research database (e.g., ProQuest, EBSCO*host*)

### Study selection and data extraction

#### Selection process

**Fig. 1:**
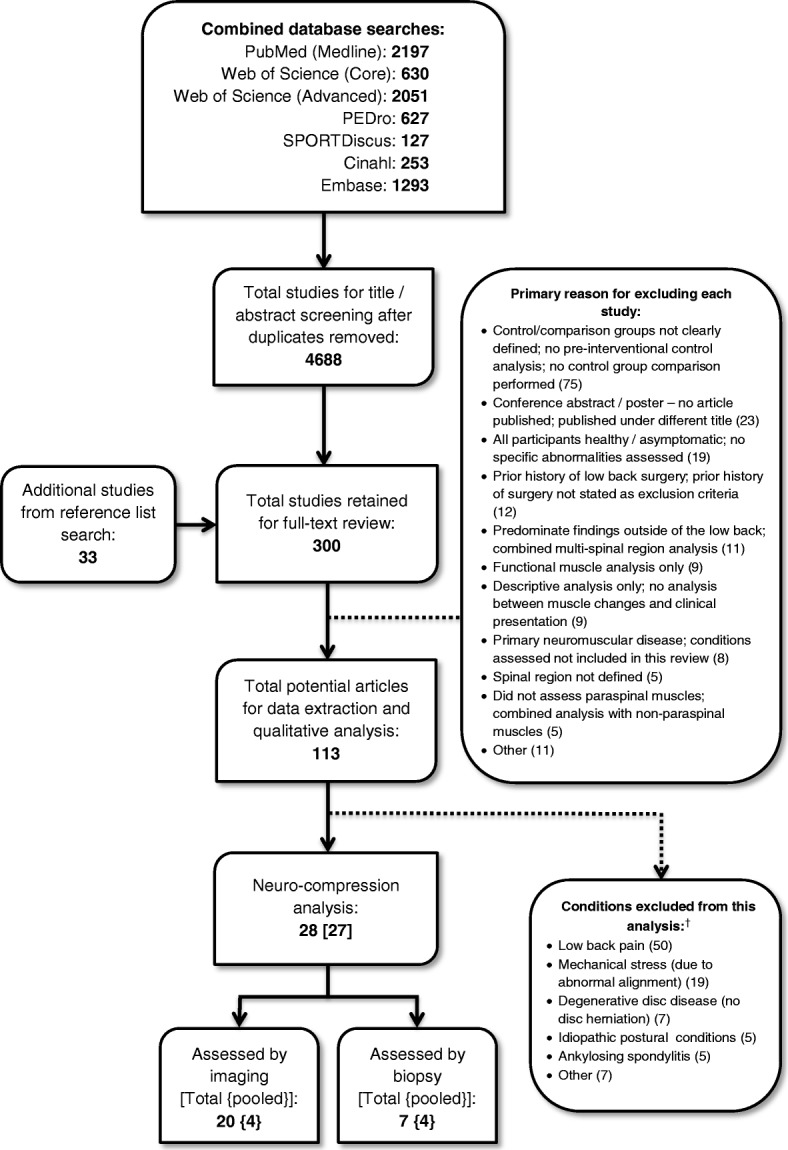
Flow diagram for search strategy. ^†^Some articles included conditions assessable in more than one subcategory

One reviewer (JC) conducted all database searches based on the previously defined strategies and removed all duplicates (Figure 1). Two review authors (JC/EA) independently screened all included titles & abstracts according to the eligibility criteria, and articles denoted as potentially eligible by *either* reviewer (i.e., “yes” or “maybe”) were included for the full-text screening stage. Articles were excluded if both reviewers indicated “no”. As there were no language restrictions applied to the search, all non-English articles selected for full-text review were professionally translated [Straker Translations (Melbourne, Victoria; Australia)].

Four reviewers participated in the full-text screening phase (JC reviewed all articles in conjunction with either EA, BW, or JH to ensure each article was initially independently assessed by two reviewers). A selection form (developed using EpiData Manager v2.0.4.43 [EpiData Association, Denmark]) was developed and piloted on ten citations, then modified for clarity (Additional File 2). Once trained, viewers assessed full-text copies of the selected articles according to the selection criteria. For full article inclusion, both reviewers of an article had to note “yes”. For exclusion, both reviewers had to indicate “no” and the recorded reason(s) agreed. Any disagreement or uncertainty regarding a decision at this stage of the process which could not be resolved by the two reviewers was presented to a third review author (i.e., an author not involved in the initial full-text review of the article) for final determination.

#### Extraction process

A data extraction form was developed using EpiData Manager (v2.0.4.43 – EpiData Association, Denmark) and pre-tested by the lead reviewer. All reviewers underwent training in the use of the form, which resulted in minor modifications to enhance clarity. Pairs of review authors independently extracted the data. Additional file 2 provides specific details regarding the type of data extracted. When extracting data, if details were not specified in the methods or results sections, “not included” was input by the reviewers.

Following extraction, a consensus meeting was held with each pair of reviewers to ensure accuracy and agreement between reviewers. Where differences were identified, disagreements were resolved via discussion or upon consultation with a third reviewer. Additionally, to identify studies potentially reporting duplicate data once the extracted data was tabulated, the lead reviewer cross-checked the study authors, year of publication, dates of data acquisition (if provided), study aims, participant demographics, methods for assessing muscle changes, and outcomes being analysed. Inter-reviewer agreement was examined by percentage agreement and Kappa coefficients, using IBM SPSS Statistics v24.0 [Armonk, NY: IBM Corp.].

### Risk of bias assessment

At the time this review was initiated, no established or validated risk of bias (ROB) tool appropriate for the types of studies predominately being assessed in this review was available. As such, we developed a modified version of the ROB tool developed by Downs and Black [28]. With one exception, the modifications applied were limited to removal of questions relating to interventions (following the approach used by Mills et al. [29], and Munn et al. [30]), and the replacement of guidance scenarios to better match the focus of our review. One reporting criterion assessing for clear descriptions of *interventions* was replaced with a criterion assessing for clear descriptions of *assessment parameters*, to include an otherwise absent key component of this review.

The three overarching criteria for assessing studies included: 1) reporting characteristics (e.g., aims, methods, participant characteristics, confounders, probability values); 2) external validity (e.g., population representation, blinding, appropriateness of analysis); and, 3) internal validity (e.g., recruitment, adjusting for confounders). Additional file 3 details the ROB tool, including more detailed explanations of the modifications applied.

The modified ROB tool for this review was piloted with each review author using three articles. Five authors independently assessed study quality (JC assessed all selected studies; EA, BW, JH, and PK assessed one or more subcomponents ensuring two independent quality assessments of each study). Disagreements within each reviewer pairing were discussed and resolved by consensus. A third reviewer was available to resolve irreconcilable differences, but this was not required. When analysing the quality of data, one assumption was made regarding the reporting of blinding: if no indication was discernible from a study’s methodology that the investigator(s) extracting clinical data were different from those assessing the imaging, it was considered that no blinding occurred between the clinical and imaging data acquisitions.

Due to the inherent issue of variable item weighting when using scaled/score-based ROB tools, an *a priori* decision was made to apply the study quality criteria used by Munn et al. [30], of <60% (low quality / high risk), 60-74% (moderate quality / moderate risk), and ≥75% (high quality / low risk) for determining overall study quality. While other studies have set a score of 50% as a quality exclusion criteria (e.g., Mills et al. [29]), we agreed with Munn that 60% was fair in our context; studies of low quality were not excluded from analysis, but their potential for increased risk of bias was considered and discussed where applicable. Inter-reviewer reliability of risk of bias was examined by percentage agreement and Kappa coefficients.

### Summary measures

For data where meta-analysis was possible, the reported means and standard deviations were used to calculate standardised mean differences (SMD). The SMD was used to allow for direct comparison of pooled results between the different continuous measurement metrics reported in our included studies, as well as to compare different constructs between analyses (e.g., measured area versus ratios or percentages). For non-pooled data, the reported measures were retained and analysed descriptively.

### Methods of analysis

For this review we undertook qualitative and quantitative analysis. For quantitative analysis, after evaluating the study outcomes for clinical homogeneity, we performed a random-effects meta-analysis on the included studies, assessing for statistical heterogeneity using both χ^2^ and I^2^ statistics. The SMD (95% CI), calculated with Hedges’ *g*, was used to report parameter estimates.

Criteria to assess clinical homogeneity between studies included patient source, sex, age, chronicity of symptoms related to neurocompression, type of comparison, imaging or biopsy method, muscle parameters assessed, and outcome scales. Meta-analyses were undertaken when three or more homogeneous studies were available. As the study effect sizes were collected from a distribution of variable effect sizes, the random-effects model was applied. Statistical analyses were conducted using Review Manager (RevMan) v5.3 [Copenhagen: The Nordic Cochrane Centre, The Cochrane Collaboration, 2014.].

### Additional analyses

Pre-specified subgroup analyses comprised disc herniations and studies with low risk of bias. A post-hoc decision was made to assess for differences in outcomes between muscle biopsy sites located at and below the level of disc herniation.

The percentage difference in muscle fiber diameter between the affected and unaffected sides of patients with LDH was calculated as the average mean diameter on the affected side / average mean diameter on the unaffected side x 100. The “average mean diameter” (AMD) per side was determined by the formula: AMD = [(m*N (S1)) + (m*N (S2)) + (m*N (S3)) + (m*N (S4))] / Total N [*S = study*].

## Results

### Study selection

The database searches identified 7178 studies, with 2490 being duplicates (Figure 1). A total of 267 studies were selected from the title/abstract search, and an additional 33 studies were identified from the reference list search of all selected articles and relevant reviews. Nine non-English language articles were included (Chinese (5), Turkish, Portuguese, Japanese, and German), of which eight met the requirements for full-text review and were fully translated. The number of studies excluded (with primary reasons indicated) at the full-text screening stage is noted in Figure 1. A list of excluded studies from the full-text phase is provided in Additional file 4.

Twenty-three potential studies for inclusion were initially identified as abstracts-only from conference proceedings or poster presentations. Upon further investigation, four of these were published under a different title and were already included for review. Authors of 15 additional abstracts were contacted with a request to confirm if their study had proceeded to full publication. Eight authors replied to either an initial or follow-up request; of these, seven indicated no publication had occurred and one provided publication details under a different title already included. No contact details for any of the authors listed for four of the abstracts could be identified. No additional studies were added from this process.

There were 113 studies initially identified for potential data extraction, of which 28 focussed on conditions relating to neurocompression. The remaining studies were excluded from this report (Figure 1), but will be considered for future systematic reviews. Of the studies identified for extraction, two [31, 32] were noted to provide different analyses of the same data set and were combined, reducing the number of distinct studies to 27. Two additional studies were published by the same lead author drawing patients from the same facility [22, 33]; however, there were sufficient differences in the methodology and patient demographics to consider these as distinct studies.

For the full-text screening phase, we achieved moderate inter-rater agreement (κ ≥ 0.68) [34] (Table 2). A third reviewer was only required on one occasion to clarify the presence of a control group.

**Table 2:** Full text screening and risk of bias agreement

Agreement for full text screening				
	Examiners 1 & 2	Examiners 1 & 3	Examiners 1 & 4	Overall
N (articles)	126	65	88	279
% agreement	83%	88%	86%	86%
κ [CI (95%)]	0.68 [0.53-0.80]	0.75 [0.58-0.91]	0.73 [0.57-0.86]	0.71 [0.63-0.80]
Agreement for risk of bias analysis				
ROB section	Reporting	External Validity	Internal Validity	Overall
N (questions)^a^	224	168	84	476
% agreement	83%	81%	73%	81%
κ [CI (95%)]	0.51 [0.38-0.63]	0.63 [0.52-0.74]	0.43 [0.28-0.62]	0.58 [0.51-0.65]

*κ* Kappa coefficient, *CI* confidence intervals, *N* number of questions

^a^Based on number of questions asked per section x 28 articles selected for neurocompression subgroup

### Study characteristics

Additional file 5 provides specific extracted participant characteristics and study details. Patients with lumbar disc herniation (LDH) were assessed via imaging in 12 studies [15, 16, 18, 35–43] and via biopsy in six studies [22, 33, 44–47], with one additional study [48] assessing subjects en bloc via biopsy across multiple pathologies with or without nerve root involvement (LDH being most frequent). Using MR or CT imaging, three studies assessed patients with facet arthrosis [49–51], four studies assessed patients with canal stenosis [20, 52–54], and two studies assessed both facet arthrosis and canal stenosis [31, 32]. These latter two studies (although eventually combined for analysis) reported outcomes separately for arthrosis and stenosis, allowing data to be assessed for each condition.

### Risk of bias within studies

During initial risk of bias analysis, overall inter-rater agreement was weak (Table 2). However, complete agreement was reached on all items during the first consensus meeting, without the need for third reviewer arbitration.

The risk of bias indices showed a wide variation in potential study bias (Table 3). Studies utilizing imaging methods to assess muscle changes tended to show lower risk of bias than those using biopsy [13.9/19 (imaging) versus 12.1/19 (biopsy)].

**Table 3:** Risk of bias index

Article	Reporting^a^								External Validity^b^						Internal Validity^b^			
	1. Study hypothesis/aim/objective clearly described?	2. Main outcome measures clearly described?	3. Characteristics of included patients clearly described?	4. Principal assessment parameters clearly described?	5. Distributions of principal confounders per group clearly described?	6. Main study findings clearly described?	7. Provides estimates of random variability for main outcomes?	10. Actual probability values reported?	11. Subjects asked to participate represent entire recruited population?	12. Subjects prepared to participate represent entire recruited population?	15. Attempt made to blind those measuring main outcomes?	16. Any results based on data dredging made clear?	18. Statistical tests were appropriate?	20. Main outcome measures accurate?	21. Cases / controls recruited from same population?	22. Cases / controls recruited over same time period?	25. Adequate adjustment for confounding?	Total score (19)
Kalichman [31]^c^	Y (1)	Y (1)	Y (1)	Y (2)	P (1)	Y (1)	Y (1)	Y (1)	Y (1)	Y (1)	Y (1)	Y (1)	Y (1)	Y (1)	Y (1)	Y (1)	Y (1)	18
Kalichman [32]^c^	Y (1)	Y (1)	Y (1)	Y (2)	P (1)	Y (1)	Y (1)	Y (1)	Y (1)	Y (1)	Y (1)	Y (1)	Y (1)	Y (1)	Y (1)	Y (1)	Y (1)	18
Kim [16]	Y (1)	Y (1)	Y (1)	Y (2)	Y (2)	Y (1)	Y (1)	Y (1)	Y (1)	U (0)	Y (1)	Y (1)	Y (1)	Y (1)	Y (1)	Y (1)	Y (1)	18
Battie [15]	Y (1)	Y (1)	Y (1)	Y (2)	Y (2)	Y (1)	Y (1)	Y (1)	U (0)	U (0)	Y (1)	Y (1)	Y (1)	Y (1)	Y (1)	Y (1)	Y (1)	17
Farshad [18]	Y (1)	Y (1)	Y (1)	Y (2)	Y (2)	Y (1)	Y (1)	Y (1)	U (0)	U (0)	Y (1)	Y (1)	Y (1)	Y (1)	Y (1)	Y (1)	Y (1)	17
Fortin [41]	Y (1)	Y (1)	Y (1)	Y (2)	Y (2)	Y (1)	Y (1)	Y (1)	U (0)	U (0)	Y (1)	Y (1)	Y (1)	Y (1)	Y (1)	Y (1)	Y (1)	17
Altinkaya [35]	Y (1)	Y (1)	Y (1)	Y (2)	Y (2)	Y (1)	N (0)	Y (1)	U (0)	U (0)	Y (1)	Y (1)	Y (1)	Y (1)	Y (1)	Y (1)	Y (1)	16
Jiang [53]	Y (1)	Y (1)	Y (1)	Y (2)	Y (2)	Y (1)	Y (1)	Y (1)	U (0)	U (0)	U (0)	Y (1)	Y (1)	Y (1)	Y (1)	Y (1)	Y (1)	16
Kalichman [49]	Y (1)	Y (1)	Y (1)	Y (2)	N (0)	Y (1)	Y (1)	Y (1)	Y (1)	U (0)	Y (1)	Y (1)	Y (1)	Y (1)	Y (1)	Y (1)	Y (1)	16
Abbas [52]	Y (1)	Y (1)	Y (1)	Y (2)	Y (2)	Y (1)	Y (1)	Y (1)	U (0)	U (0)	U (0)	Y (1)	Y (1)	Y (1)	Y (1)	N (0)	Y (1)	15
Boyaci [36]	Y (1)	Y (1)	Y (1)	Y (2)	P (1)	Y (1)	Y (1)	Y (1)	U (0)	U (0)	N (0)	Y (1)	Y (1)	Y (1)	Y (1)	Y (1)	Y (1)	15
Zhao [47]	Y (1)	Y (1)	Y (1)	Y (2)	Y (2)	Y (1)	Y (1)	N (0)	U (0)	U (0)	U (0)	Y (1)	Y (1)	Y (1)	Y (1)	Y (1)	Y (1)	15
Bhadresha [40]	Y (1)	Y (1)	Y (1)	Y (2)	P (1)	Y (1)	Y (1)	Y (1)	U (0)	U (0)	U (0)	Y (1)	Y (1)	Y (1)	Y (1)	Y (1)	N (0)	14
Mattila [46]	N (0)	Y (1)	Y (1)	Y (2)	P (1)	Y (1)	Y (1)	N (0)	Y (1)	Y (1)	U (0)	Y (1)	Y (1)	Y (1)	Y (1)	U (0)	Y (1)	14
Ogon [54]	Y (1)	Y (1)	Y (1)	Y (2)	Y (2)	Y (1)	Y (1)	Y (1)	U (0)	U (0)	U (0)	Y (1)	Y (1)	Y (1)	U (0)	U (0)	Y (1)	14
Sebro [50]	Y (1)	Y (1)	Y (1)	Y (2)	Y (2)	N (0)	N (0)	Y (1)	U (0)	U (0)	U (0)	Y (1)	Y (1)	Y (1)	Y (1)	Y (1)	Y (1)	14
Yarjanian [20]	Y (1)	Y (1)	Y (1)	P (1)	P (1)	Y (1)	Y (1)	Y (1)	U (0)	U (0)	Y (1)	Y (1)	Y (1)	U (0)	Y (1)	Y (1)	Y (1)	14
Yoshihara [22]	Y (1)	Y (1)	N (0)	Y (2)	Y (2)	Y (1)	Y (1)	N (0)	U (0)	U (0)	U (0)	Y (1)	Y (1)	Y (1)	Y (1)	Y (1)	Y (1)	14
Yu [51]	Y (1)	Y (1)	Y (1)	P (1)	P (1)	Y (1)	Y (1)	Y (1)	U (0)	U (0)	Y (1)	Y (1)	U (0)	Y (1)	Y (1)	Y (1)	Y (1)	14
Hyun [38]	Y (1)	Y (1)	Y (1)	Y (2)	P (1)	Y (1)	Y (1)	N (0)	U (0)	U (0)	Y (1)	Y (1)	Y (1)	Y (1)	Y (1)	U (0)	N (0)	13
Yoshihara [33]	Y (1)	Y (1)	Y (1)	Y (2)	Y (2)	N (0)	N (0)	N (0)	U (0)	U (0)	U (0)	Y (1)	Y (1)	Y (1)	Y (1)	Y (1)	Y (1)	13
Ford [45]	N (0)	Y (1)	N (0)	Y (2)	P (1)	Y (1)	Y (1)	N (0)	U (0)	U (0)	U (0)	Y (1)	Y (1)	Y (1)	Y (1)	Y (1)	Y (1)	12
Frost [42]	Y (1)	Y (1)	Y (1)	Y (2)	Y (2)	N (0)	Y (1)	N (0)	U (0)	U (0)	U (0)	Y (1)	Y (1)	Y (1)	U (0)	U (0)	Y (1)	12
Dangaria [37]	Y (1)	Y (1)	Y (1)	P (1)	P (1)	Y (1)	N (0)	N (0)	U (0)	U (0)	U (0)	Y (1)	Y (1)	Y (1)	Y (1)	U (0)	Y (1)	11
Kong [39]	Y (1)	Y (1)	Y (1)	P (1)	Y (2)	Y (1)	Y (1)	N (0)	U (0)	U (0)	U (0)	Y (1)	Y (1)	U (0)	U (0)	U (0)	Y (1)	11
Bajek [44]	Y (1)	Y (1)	N (0)	Y (2)	P (1)	Y (1)	Y (1)	N (0)	U (0)	U (0)	U (0)	Y (1)	Y (1)	Y (1)	U (0)	U (0)	N (0)	10
Sun [43]	Y (1)	N (0)	Y (1)	P (1)	P (1)	Y (1)	N (0)	N (0)	U (0)	U (0)	U (0)	Y (1)	Y (1)	Y (1)	Y (1)	Y (1)	U (0)	10
Jowett [48]	Y (1)	Y (1)	N (0)	Y (2)	P (1)	N (0)	N (0)	N (0)	U (0)	U (0)	U (0)	Y (1)	U (0)	Y (1)	U (0)	U (0)	N (0)	7
Total “Yes” (28)	26	27	24	23	14	24	22	16	5	3	11	28	26	26	23	19	23	

^a^For items 4 & 5, articles that provided some but not all relevant criteria were rated as “partial”. For item 10, articles were rated “yes” if actual values were provided for the majority of reported outcomes

^b^For items 11-25, articles were rated as “unable to be determined” if insufficient details were provided to make a determination. For item 15, articles were rated as “unable to be determined” if it was unclear regarding blinding when the same investigators were involved in obtaining the pathology details and performing the muscle evaluations; this was also the case when it was unclear if the clinical pathology and muscle assessments were performed by the same investigators

^c^These studies appeared to use different methodologies to analyse the same dataset so were combined for final analysis

### Risk of bias across studies

**Fig. 2:**
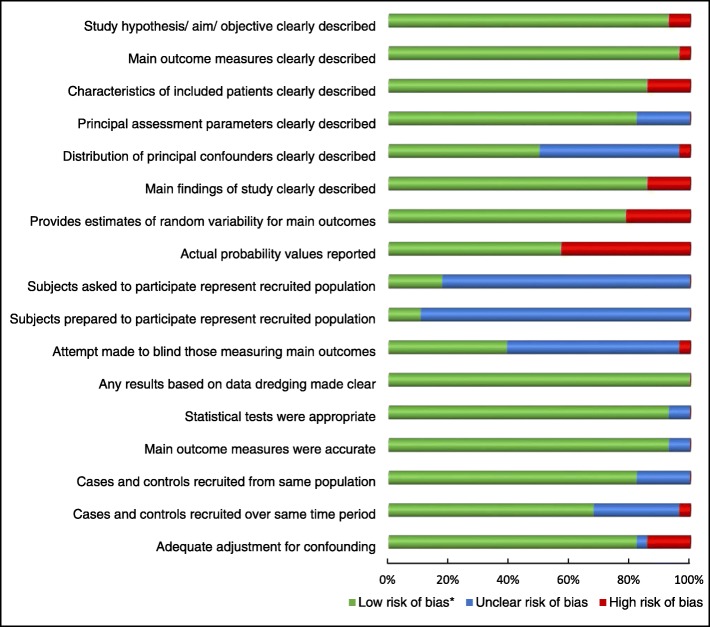
Risk of bias across studies. *Low risk of bias: ROB tool criteria = Yes; Unclear risk of bias: ROB tool criteria = Partial or Unable to be determined; High risk of bias: ROB tool criteria = No

Figure 2 provides a graphic breakdown of potential bias across studies. The four areas of risk most consistently identified related to: a) uncertainty regarding recruited population representation, with most studies failing to provide sufficient descriptive data to make a determination; b) lack of reporting of actual probability values, with newer studies more likely to provide these values; c) distribution of principal confounders, with nearly half the studies providing only partial details; and d) blinding. An inherent blinding challenge existed for the imaging-based studies – even if the examiner measuring the muscles was blinded to the imaging pathology report, the pathology would most likely be evident on the images if it was not specifically blocked from view.

### Study findings with syntheses of results

For each of the following sections, a compilation of the relevant outcome details for the included studies is provided in Additional file 5. Attempts were made via email to contact authors when issues with study data required clarification. In two cases details were not obtained, one reporting anatomically improbable measurement ranges for some data [37], and one with missing error values for some outcomes [33]. In both instance these data were removed from analysis.

#### Paraspinal muscle morphology in patients with lumbar disc herniation (LDH) – assessed with imaging

##### Study characteristics and ROB

Twelve studies assessed patients with unilateral LDH with radiculopathy; 11 using MRI and one using diagnostic ultrasound. Of these, six had a low risk of bias, three a moderate risk, and three a high risk; total sample sizes varied from 33 to 165 participants. In four studies, patients served exclusively as their own controls (involved vs uninvolved sides) [15, 18, 35, 41], two studies used both patients and healthy volunteers as controls [37, 42], and one study used the patients and healthy volunteers as controls plus included an LDH group without radiculopathy as a comparison [38]. One study compared acute versus chronic radiculopathy patients as well as using patients in each group as their own controls [16], while another study used healthy participants as the only control [39]. Two studies used low back pain patients without LDH or nerve root compression as a comparison – one chronic [36] and one non-specific [43], and the final study used chronic low back pain patients with degenerative disc disease (DDD) without LDH as a comparison [40]. All but one study assessed the lumbar multifidus muscles (LMM) (with or without including the erector spinae muscles (ESM)) and four studies included the psoas major muscles (PMM). Multiple measures of muscle morphology were used in most studies, with the total cross-sectional area (TCSA) and/or functional cross-sectional area (FCSA) being most consistently assessed.

##### Meta-analysis

**Fig. 3:**
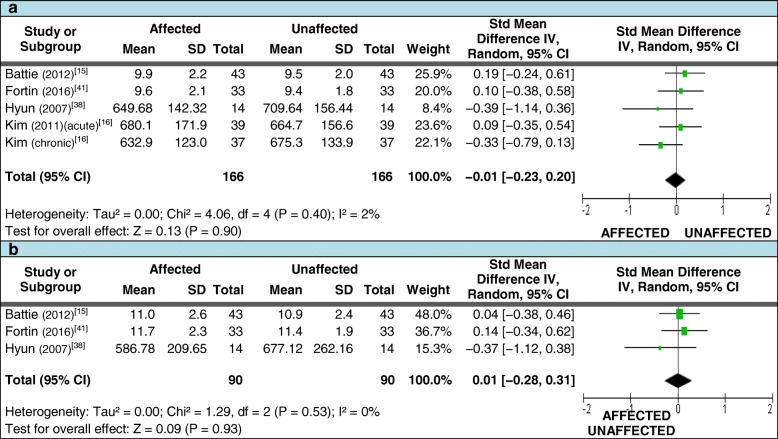
Pooled LMM imaging measurements – TCSA**.** Pooled total cross-sectional area (TCSA) measures for meta-analysis comparing the side affected by disc herniation to the unaffected side. 3**a**: at the level of herniation; 3**b**: below the level of herniation

Four studies met our criteria for pooled data analysis assessing for differences in mean LMM TCSA [15, 16, 38, 41] (refer to Additional file 5 for study details). For those measures taken at the level of LDH, 166 patients with unilateral LDH where included but demonstrated no significant difference in the pooled SMD between sides (Figure 3a). A total of 90 patients were included for measurements below the level of LDH, also showing no differences (Figure 3b). As there were diverse outcomes between studies, subgroup analyses were undertaken to determine if this was dependent on the duration of symptoms; however, the pooled SMD remained non-significant [at the level of LDH – only acute included [0.14 (95% CI = −0.16, 0.45] and acute excluded [−0.17 (95% CI = −0.47, 0.14)]; below the level of LDH – only acute included [0.04 (95% CI = −0.38, 0.46)] and acute excluded [−0.03 (95% CI = −0.50, 0.44)]].

**Fig. 4:**
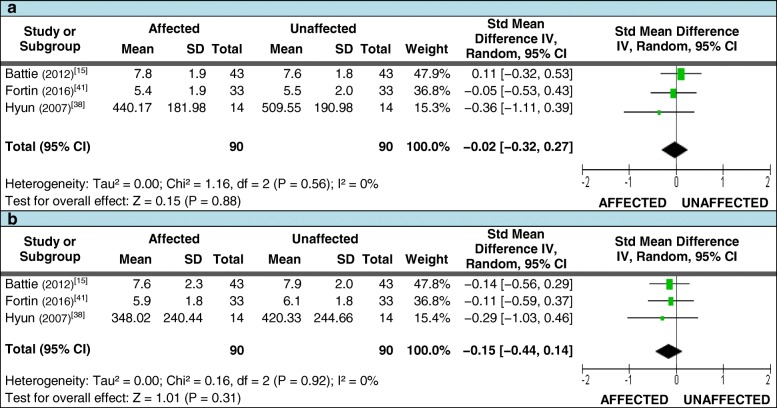
Pooled LMM imaging measurements – FCSA. Pooled functional cross-sectional area (FCSA) measures for meta-analysis comparing the side affected by disc herniation to the unaffected side. 4**a**: at the level of herniation; 4**b**: below the level of herniation

**Fig. 5:**
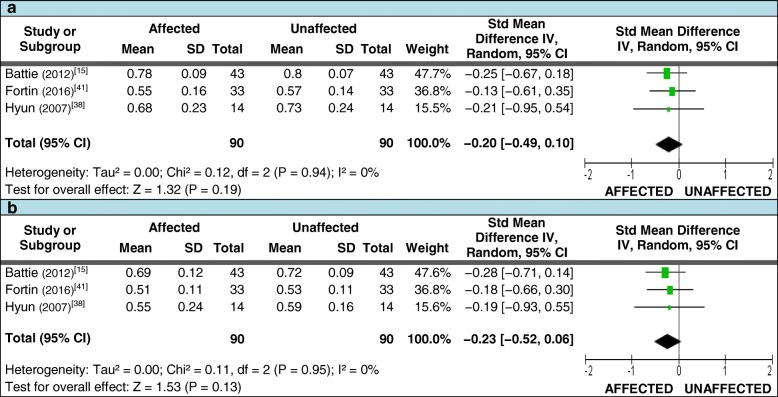
Pooled LMM imaging measurements – FCSA:TCSA ratio. Pooled FCSA:TCSA ratio measures for meta-analysis comparing the side affected by disc herniation to the unaffected side. 5**a**: at the level of herniation; 5**b**: below the level of herniation

Three of these studies also met the criteria for assessing differences in the mean FCSA and FCSA:TCSA ratios [15, 38, 41]. For FCSA measures taken at or below the level of LDH, 90 patients with unilateral LDH where included; the pooled SMD again demonstrated no difference between sides (Figures 4a and 4b). A total of 90 patients were also included for FCSA:TCSA ratio measures at and below the level of LDH. While all studies demonstrated smaller mean measures on the affected side at both levels, no significant difference in the pooled SMD between sides was found (Figures 5a and 5b). Table 4 (section 1.0) reports the qualitative synthesis results from relevant studies not included in the meta-analyses.

**Table 4:** Detailed results analysis for non-pooled data

1.0 Paraspinal muscle morphology in patients with lumbar disc herniation – assessed with imaging:	
1.0.1 Patients serving as own controls • 3 studies assessed the TCSA of the PMM [15, 16, 37]: ○ [15]: acute patients: PMM was larger on side of LDH regardless of measurement relationship to LDH; not statistically significant. ○ [16]: acute and chronic patients at LDH level only: PMM CSA was insignificantly smaller on side of LDH for both groups. ○ [37]: chronic patients: PMM was smaller on side of LDH regardless of relationship to level of LDH; statistically significant at L4/5, L5/S1 (P < 0.05). Median % reduction of TCSA on side of LDH averaged 8.5% (*P* < 0.05) at L4/5, L5/S1. • 2 high quality studies assessed the TCSA and FCSA of the ESM [15, 41]: ○ [15]: no significance to these differences at any level. ○ [41]: significantly smaller FCSA measures at L5/S1 (level below LDH) (*P* = 0.04), and significantly smaller ratios at L4/5, L5/S1 (*P* = 0.04, 0.007); TCSA on side of LDH was larger at the level above LDH (*P* = 0.05). • 2 studies assessed MRI signal intensity of the LMM and ESM: one including acute [15], one chronic [41] patients. Both studies demonstrated: ○ significant increase in mean LMM signal (i.e., more fat) on side of LDH at the level below (*P* = 0.014 [15] and 0.04 [41]); no consistent or significant differences noted at or above the level of LDH. ○ higher mean ESM signal on side of LDH at the level of LDH in acute patients (*P* = 0.017) and the level below LDH (S1) in chronic (*P* = 0.02). • 1 moderate quality study assessed the combined echo intensity of the LMM and ESM [42], and reported statstically non-significant results. • 1 high quality study compared the affected to non-affected side to assess median TCSA and MLD of the LMM against duration of symptoms and severity of NR compression [35], reporting: ○ non-significant results for TSCA. ○ MLD larger on side of LDH across all *duration* groups (P < 0.05); MLD progressively enlarged as duration increased (P = 0.021). ○ non-significant results for MLD across all *severity* groups. • 1 high quality study assessed FCSA and MLD ratios of the LMM, and their relationship to various clinical measures [18]: ○ no significant relationship found between FCSA or MLD ratios and severity of NR compression, symptom duration, or motor deficit. ○ 1 moderate quality study assessed TCSA, FCSA, and FCSA:TCSA ratios in LDH patients without radiculopathy [38], reporting no significant differences. 1.0.2 Compared to healthy controls without LDH or radiculopathy • 4 LDH studies included a healthy control group [37–39, 42]: ○ [37]: measured side-to-side difference in TCSA of the PMM in a control group: no difference found between sides. Control group median TCSA was smaller than both LDH groups from L3/4 – L5/S1, but no statistical comparison was made between groups. ○ [38]: compared TCSA, FCSA, FCSA:TCSA ratio, and involved:uninvolved side FCSA ratios (IS:US) of control group LMM to patients with LDH – with and without radiculopathy. ▪ TCSA smaller at L5/S1 on side of LDH in both patient groups (*P* < 0.05); FCSA significantly smaller in both patient groups at L4/5, L5/S1 (*P* < 0.05). ▪ FCSA:TCSA ratio smaller in both patient groups at L3/4, L4/5 (*P* < 0.05). ▪ IS:US ratio for radiculopathy group significantly smaller than controls at L4/5, L5/S1 (*P* < 0.01), and when all levels were combined (*P* < 0.05). ▪ IS:US ratio abnormal in 79% of radiculopathy cases and 10% of controls (*P* < 0.01), but not between control group and uninvolved side of LDH. ○ [39]: assessed amount of combined fat infiltration of LMM and ESM (presumably bilaterally, but not defined). Fat infiltration was greater in the LDH group at all levels (*P* < 0.05 at L2/3; *P* < 0.001 from L3/4 – L5/S1). ○ [42]: assessed echo intensity of LMM and ESM combined, with no difference noted between any groups. 1.0.3 Compared to low back pain patients without LDH or radiculopathy • 3 LDH studies included LBP comparison groups; 1 high quality [36], 1 moderate quality [40], and 1 low quality [43]: ○ [36]: compared TCSA and quantitative gradings of LMM, PVM, PMM, QLM for LDH with unilateral or bilateral radiculopathy to chronic LBP only patients. ▪ smaller TCSA of right QLM only noted in the LDH group (*P* = 0.01). ▪ higher grades of fat infiltration more prevalent in LDH group at all locations except PMM (*P* range: 0.02 – 0.04). ▪ NB: 8 patients in the comparison group also had facet arthrosis, but no leg pain. ○ [40]: used point-of-contact calculations (Cavalieri approximation principle) to compare muscle:fat ratios of LMM, ESM, & PMM in single or multi-level LDH patients to same ratios in LBP patients (unknown symptom duration) with single or multi-level degenerative disc disease; individual muscles were combined bilaterally. No difference in ratios found between patient groups for any muscle at any level. ○ [43]: used quantitative muscle grading to assess the LMM bilaterally from L3/4 – L5/S1; greater atrophy, and more severe atrophy, reported in LDH group at all levels (*P* < 0.01). NB: no analyses made regarding side of LDH; unknown if differences in symptom chronicity present requiring adjustment. 1.0.4 Compared to LDH patients without radiculopathy • 1 study compared the TCSA, FCSA, FCSA:TCSA ratio, and IS:US ratio (FCSA) of the LMM between LDH patients with and without radiculopathy [38]: ○ IS:US ratio for radiculopathy group smaller than LDH-only group at L4/5, L5/S1 (*P* < 0.01), and with all levels combined (*P* < 0.05). ○ IS:US ratio abnormal in 24% of LDH-only cases vs. 79% of radiculopathy cases (*P* < 0.01). ○ no differences in the remaining CSA measures were noted.	
1.1 Paraspinal muscle morphology in patients with lumbar disc herniation – assessed with biopsy:	
• 3 studies assessed mean fiber type diameter and distribution for the ESM [45] and the LMM [44, 46]: ○ [45]: compared the affected to non-affected side and found no difference for any measures. ○ [44]: compared to deceased controls: Type I fiber distribution in LMM higher for males with LDH (P<0.05); type I fiber diameter larger in males (P<0.05) and females (*P* < 0.01) with LDH; type IIA and IIB fiber diameter larger in male LDH patients (*P* < 0.05). NB: not adjusted for differences in age or sex. ○ [46]: compared to deceased controls: Type I fiber diameter was significantly larger in male LDH patients (*P* < 0.01). • 2 studies assessed mean muscle strength factor (MSF), one for the LMM and ESM [45], and one for the LMM only [47]: ○ [45]: non-significant results for both muscle groups. ○ [47]: type II fiber MSF was lower on the LDH side (*P* < 0.05). • 2 studies compared % frequencies of fiber type grouping and small angular fibers in the LMM [22, 33]: ○ [22]: higher grouping frequency on side of LDH at the level below LDH (L5): 27.6% vs.10.3%; higher angular fiber frequency noted on side of LDH at L5: 20.7% vs. 3.4% (no P values). ○ [33]: higher grouping frequency on side of LDH at L5 (level below LDH): 35% vs. 6%; higher angular fiber frequency noted on side of LDH at L5: 41% vs. 24% (no *P* values). • 1 study, using patients as their own control, measured mean fiber type CSA of the LMM, and fiber CSA with or without a +SLR [47], noting: ○ significantly and consistently smaller CSA for both fiber types on affected side of LDH (*P* < 0.05); this became more pronounced when considering +SLR patients only (*P* < 0.01), but non-significant with −SLR patients only. • 1 moderate quality study assessed mean atrophy/hypertrophy factors for type I & II fibers, and mean % core targetoid and moth-eaten change in the LMM against cadaveric controls [46]: ○ an increase in core-targetoid presence was found in male (*P* < 0.01) and female patients (*P* < 0.001), with higher type I fiber hypertrophy factor in male patients (*P* < 0.01) and an increase in moth-eaten change in female patients (*P* < 0.001).	
1.3 Paraspinal muscle morphology in patients with facet arthrosis – assessed with imaging:	
• 4 studies assessed the association of facet arthrosis with paraspinal muscle density (2 using the same general population data set) [(31,32),49,50]: ○ [31]: reported severe arthrosis (grade 3) at L4/5 consistently associated with greater fat infiltration of the LMM and ESM (*P* range: 0.0002 – 0.056). ○ [32]: identified associations between reduced paraspinal muscle density and arthrosis [AOR: 3.68 [1.36 – 9.97] (LMM); 2.80 [1.10 – 7.16] (ESM)]. ○ [49]: assessed L4/5, with AORs showing associations between arthrosis and muscle density ratios (*P* range: 0.001 – 0.009 (LMM); 0.002 – 0.01 (ESM)), as well as arthrosis and higher fat infiltration grades (*P* <0.0001 (LMM & ESM)). No associations found between arthrosis and mean muscle density only. ○ [50]: demonstrated negative correlations between LMM, PMM, and Longissimus muscle density and facet arthrosis with univariate analysis (*P* <0.0001 for all muscles), but non-significant correlations following multivariate analysis. ▪ NB: this study grouped all measures rather than assessing muscle density or arthrosis by spinal level. • 1 study assessed arthrosis in relation to CSA and the muscle-fat index (MFI) [51], identifying: ○ smaller CSA and higher MFI (i.e., higher fat content) at all levels with arthrosis present (*P* < 0.001); increased MFI was independently associated with arthrosis at all levels (*P* range: <0.001 – 0.005). ○ differences in CSA asymmetry were greater for CSA with arthrosis present at L5/S1 only (*P* range: <0.001 – 0.005).	
1.4 Paraspinal muscle morphology in patients with canal stenosis – assessed with imaging:	
• 4 studies assessed the association of canal stenosis with paraspinal muscle fat inflitration (2 using the same general population data set) [(31,32),52,53]: ○ [31, 32]: after adjusting for age, sex, and/or BMI, spinal stenosis was not associated with altered CT muscle density for the LMM or ESM (N = 15). ○ [52]: showed associations of stenosis with increased CT muscle density in LMM, ESM, and PMM in 165 patients with confirmed clinical symptoms of stenosis (*P* range = 0.036 to < 0.001). ▪ NB: the lower fat content in the stenosis group may be the result of the muscles being measured above the level of reported stenosis. ○ [53]: assessed fat infiltration with MRI at the level of stenosis, with a greater fat infiltration ratio in the stenosis group (N = 40; *P* = 0.004). • 3 studies assessed the relationship of stenosis to muscle atrophy [20, 52, 53]: ○ [20]: reported a reduction in FCSA of LMM between stenosis and healthy control groups (*P* = 0.04), but not stenosis and LBP groups. ○ [52]: demonstrated greater FCSA of the ESM in male (*P* = 0.011) and female (*P* = 0.014) stenosis patients, and PMM in male stenosis patients (*P* = 0.042), but no significant difference for the LMM. NB: acquired measurements at L3, which could account for conflicting results with other studies. ○ [53]: evaluated muscle asymmetry and TCSA measures of the LMM at L4/5;with greater asymmetry (*P* = 0.006) and lower TCSA:body ratios (*P* = 0.006) reported in the stenosis group. • 1 study used MR spectroscopy to assess extramyocellular lipids (EMCL) and intramyocelluar lipds (IMCL) [54]: ○ when assessing EMCL content (i.e, the fat tissue deposits visible on standard MR imaging), no difference was found between the stenosis and CLBP groups; this is in agreement with the study by Yarjanian above [20]. ○ IMCL content was higher in the CLBP vs. stenosis group (*P* =< 0.01). ▪ NB: IMCL deposits not are seen on standard imaging, being more likely to increase due to metabolic changes associated with inactivity; significantly higher VAS scores in the CLBP group would be a more important contributor than potential NR compression. ▪ NB: this study also analysed postural changes between each group but found no differences; alignment was not a confounding factor in this analysis.	

*LMM* Lumbar multifidus muscle, *PMM* Psoas major muscle, *PVM* Paravertebral (paraspinal) muscle, *ESM* Erector spinae muscle, *QLM* Quadratus lumborum muscle, *LBP* Low back pain, *SLR* Straight leg raise, *NR* Nerve root, *LDH* lumbar disc herniation, *CSA* Cross-sectional area, *TCSA* Total CSA, *FCSA* Functional CSA, *MLD* Muscle laminar distance, *IS US ratio* Involved side to uninvolved side ratio, *VAS* Visual analogue scale, *AOR* Adjusted odds ratio, *BMI* body mass index, *CT* Computed tomography, *MRI* Magnetic resonance imaging

#### Paraspinal muscle morphology in patients with lumbar disc herniation – assessed with biopsy

##### Study characteristics and ROB

Six studies assessed patients with unilateral LDH with radiculopathy using muscle biopsy. Of these, one was of high quality, four of moderate quality, and one of low quality; study sample sizes ranged from 17 – 117. In four studies, patients served exclusively as their own controls (involved vs. uninvolved sides) [22, 33, 45, 47], and in two studies recently deceased persons who were previously healthy served as the control group [44, 46]. All studies assessed the LMM, with one study [45] also assessing the ESM. Although various measurement parameters were used across studies, they all included the mean fiber type distribution and diameter.

##### Meta-analysis

**Fig. 6:**
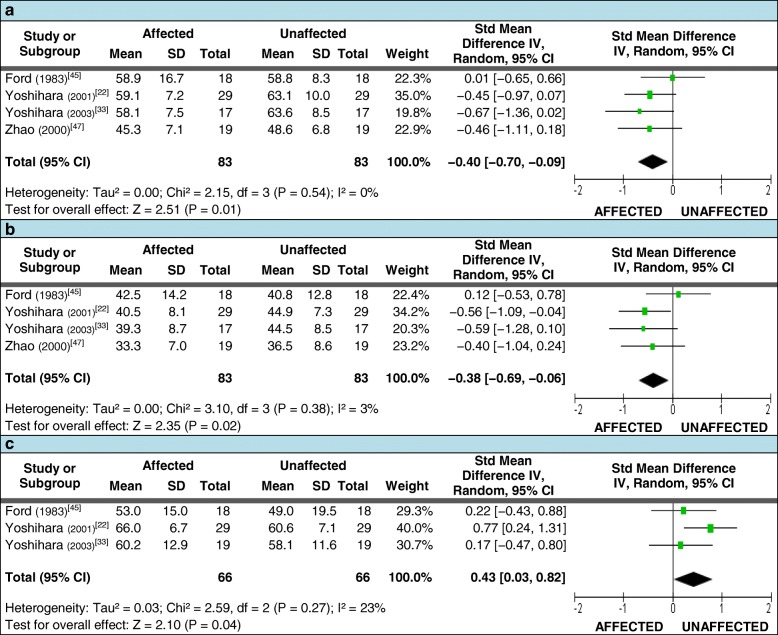
Pooled LMM biopsy measurements. Pooled biopsy measures for meta-analysis comparing the side affected by disc herniation to the unaffected side. 6**a**: type I fiber size; 6**b**: type II fiber size; 6**c**: type I fiber distribution

All four studies with patients serving as their own control met the criteria for pooled data analysis when assessing mean fiber type diameter of the LMM. These studies measured type I and II fiber diameter (μm) at a total of 112 spinal levels in 83 unilateral LDH patients undergoing surgical intervention, with each study including both acute and chronic patients (refer to Additional file 5 for additional study details). The pooled analysis demonstrated a reduction of type I fiber diameter on the side of LDH (Figure 6a), which equated to the average mean diameter being 5.5% smaller on the side of LDH; similar results were seen for type II fiber diameter (Figure 6b), with the average mean diameter being 6.8% smaller on the side of LDH. The study by Ford et al. [45], contradicted the findings of the other three studies for both fiber types, but it was the lowest quality study and provided the least details regarding the relationship of the LDH to the muscle level biopsied.

Three of the above studies also met the criteria for pooling the assessment of differences in the mean fiber type distribution [22, 45, 47]. Although none of these studies reported a significant difference in fiber distribution individually, their pooled SMD demonstrated an increase in type I fiber distribution on the side of LDH (Figure 6c), which equated to a 7% greater average mean fiber distribution. The fourth study was not included in fiber distribution pooled data analysis due to the absence of a reported variance estimate which could not be obtained from the authors; however, consistent with the pooled data it did report a higher mean distribution of type I fibers on the side of LDH [33].

For the above analyses, only the measurements at the level below herniation were used from the Yoshihara et al. (2001) study [22]; results taken at the level of LDH were also available, but rather than combining the two sets of values, the latter dataset was included in a subsequent subgroup analysis based on the level of biopsy in relation to LDH. As the study by Ford et al., did not specify this relationship, it was excluded from further analysis. For biopsies acquired at [22, 47] or below [22, 33] the level of LDH, type I and II fiber diameter measures were only smaller on the affected side for muscles below the level of LDH: type I fiber diameter at the level of LDH (SMD [95% CI] = −0.27 [−0.68, 0.13]) and below the LDH (SMD [95% CI] = −0.53 [−0.95, −0.11]); type II fiber diameter at the level of LDH (SMD [95% CI] = −0.30 [−0.71, 0.10]) and below the LDH (SMD [95% CI] = −0.57 [−0.99, −0.16]). There was insufficient data to perform subgroup analysis on fiber type distribution. Table 4 (section 1.1) shows results from the qualitative synthesis for this section.

#### Paraspinal muscle morphology in patients with any spinal pathology and associated nerve root signs – assessed with biopsy

One study assessed the distribution of LMM fiber types in patients with spinal pathology with and without signs of NR involvement, along with a cadaveric control group [48]. The limited distinction of pathology types precluded pathology-based analysis. Although a significant difference was demonstrated in the percentage of type II fibers, both measures fell within the average type II fiber distribution of ~36% (±11%) noted by Mannion et al. [55], in a young, healthy population. This was the oldest and highest risk of bias study in this systematic review (7/19).

#### Paraspinal muscle morphology in patients with facet arthrosis – assessed with imaging

##### Study characteristics and ROB

Four studies looked at three different data sets utilizing CT imaging to assess fatty infiltration of paraspinal muscles in individuals with facet arthrosis: two from the same general population [31, 32] and two from patient populations [49, 50]. Three studies were of high quality and one of moderate quality; total sample sizes varied from 100-187. Three studies assessed facet arthrosis and muscle changes at multiple spinal levels; one study evaluated the L4/5 level only [49]. Three studies evaluated the LMM and ESM and compared participants with arthrosis to those without; the remaining study assessed the LMM, PMM, and longissimus and assessed arthrosis on a summative grading scale [50]. A fifth, moderate quality study used MRI and CT to assess CSA and fatty infiltration of the LMM [51]. All studies applied different statistical analyses to the relationships between arthrosis and muscle changes, precluding data pooling. Table 4 (section 1.3) provides the results from the qualitative synthesis for this section.

#### Paraspinal muscle morphology in patients with canal stenosis – assessed with imaging

##### Study characteristics and ROB

Six studies looked at five different data sets to assess relationships between central stenosis and muscle morphology, with four being of high quality and two of moderate quality; total sample sizes ranged from 35 – 345. Two studies used the same CT data set to assess fatty infiltration of the LMM and ESM in a general volunteer population [31, 32]. The remaining studies evaluated patients with clinical and/or imaging findings consistent with stenosis; one used CT [52], two used MRI [20, 53], and one used MR spectroscopy [54]. Of these latter four studies, one compared spinal stenosis patients to LBP patients without spinal stenosis as well as asymptomatic volunteers [20], one compared stenosis to chronic LBP patients only [54], while to remaining two studies compared patients with and without stenosis only. Muscle analysis utilized a variety of approaches and statistical analyses were also quite variable, precluding the pooling of data*.* Table 4 (section 1.4) reports the results from the qualitative synthesis data for this section.

## Discussion

This systematic review is the first to synthesize studies examining the relationships between paraspinal muscle morphology and spinal pathologies associated with neurocompression in patients with specific LBP. We found LDH to be associated with muscle morphological changes comprising fiber size, fiber type, and fiber distribution. Specifically, the findings of our meta-analyses demonstrated that when patients served as their own controls, LDH was associated with decreased type I and II fiber size, and an increased proportion of type I fibers, in the LMM at the level below the herniation; this could be related to compressive nerve root damage leading to muscle fiber denervation [46]. From the qualitative synthesis of individual studies (Table 4), we found a higher frequency of small angular fibers (indicating denervation of single motor neuron muscle fibers [46]) and fiber type grouping (indicating collateral re-innervation of these fibers [46]) on the side of and below LDH, which correlated with the more significant amount of fiber atrophy found at the level below herniation in the pooled data. A higher percentage of core targetoid change was also identified at the level below LDH in one study, which is a non-specific indicator of underlying muscle disease, including denervation [46]. The findings from the pooled and non-pooled data suggest that persistent compression of the nerve roots may be contributing to atrophy of muscle fibers supplied by that nerve. Whether these changes are permanent or reversible is unclear.

Pooling of data from studies that used imaging modalities to measure the cross-sectional area of paraspinal muscles did not identify associations with spinal pathology. However, several individual studies did report associations between spinal pathology and imaging derived measures of paraspinal muscle morphology, particularly regarding LDH with chronic radiculopathy, and facet arthrosis [31, 32, 35, 36, 38, 39, 43, 49, 51]. Increased fatty infiltration of the PVM occurred with higher grades of facet degeneration, particularly at L4/5 (where facet joint arthrosis is most commonly found [56]). For central canal stenosis, the limited number of studies, conflicting outcomes, and key variations in study methodology precluded a definitive conclusion.

The absence of findings of a consistent reduction of muscle CSA in the presence of these specific pathological conditions may indicate that no significant relationships exist; however, it may also be possible that the variability in study designs is partially concealing the impact of the changes. For example, mixing measures across spinal levels rather than specifically measuring “at” and “below” the herniation, measuring above the level of pathology, grouping all spinal levels instead of individual analysis, or mixing acute and chronic back pain patients in the same analysis. Conversely, any number of study design or measurement variations could also have resulted in the apparent mismatch between the biopsy and imaging findings; however, actual morphological reasons for this difference may relate to fiber type distribution being less apparent anatomically and thus only notable with biopsy, or the internal complexity of the gross anatomy of the LMM masking microscopic changes to individual fiber size on imaging. Additionally, imaging modalities cannot provide the same level of precision as histological studies.

Potential confounders to be considered when interpreting the outcomes of this review include the neurological supply of the multifidus muscles, how muscle atrophy presents, and the types of controls used between studies. When considering uni-segmental versus multi-segmental nerve supply to the LMM, it is physiologically apparent that muscle activation (whether normal or pathological) can occur well above or below the level of primary nerve root involvement, even if the anatomical data suggests level-specific innervation [57]; however, Kottlors et al. [57], have suggested that this effect reduces the further away the level of muscle origin is from the nerve root affected. If that is the case, the primary alteration to the LMM from any nerve root lesion should be most profound at the level supplied by the medial branch of the dorsal ramus of the affected nerve root, with progressively less change occurring to the muscles farther away. This may help explain the occasional finding of reduced LMM FCSA (albeit insignificantly) of the muscle above an affected nerve root, but the greater likelihood of significant FCSA reduction of muscles supplied primarily by a compressed nerve root.

Within several studies, the side-to-side differences in the reported TCSA were less consistent than those noted for the FCSA. While muscle atrophy is most simply assessed by imaging as a reduction in the overall size of a muscle’s TCSA, this does not take into account the possibility that individual muscle fascicles may atrophy and be replaced by fat infiltration [58] without reducing the muscle’s total cross-section. This change may manifest most clearly in the multifidus muscle fascicles closest to the spinolaminar margins (as visualized on axial cross-sections from L4-S1), which are directly innervated by an affected L4 or L5 nerve root. This variability could be accounted for in the assessment of atrophy if a muscle-to-fat ratio component is included, and by including all tissue within the epimysial boundaries.

The issue of using patients as their own controls, versus healthy (with imaging studies) or cadaveric controls (for biopsy studies) was considered. The advantages of using patients as their own controls includes consistency of image parameters, quality, spinal level selection and patient parameters (e.g., matching size, age, sex variables), as well as being generally more convenient since fewer participants are required. Disadvantages include the potential for inherent confounders, such as normal asymmetry, any effect of the pathologic variable on the contralateral side, or the potential for neurological alterations contralateral to the side of pathology [57]. However, our review did not show outcomes to be greatly varied between studies based on the type of control group, except with biopsies using cadaveric controls.

## Limitations

A key challenge for undertaking this review was the inherent difficulty in assessing paraspinal muscle morphology by any study looking at these muscle groups, due to a lack of agreement on multifidus muscle gross and neuroanatomy; at least ten published variations are described. The 2008 study by Lonnemann et al. [59], provides a clear overview of these descriptions, but also offers a new one. This is further complicated by a 2011 article by Cornwall et al. [60], describing anatomy more closely found in the seminal study by Macintosh et al. [61], but with their own distinct alternations to that description. Nevertheless, the Lonnemann [59] and Cornwall [60] studies both agree on the complex inter-digitation or blending of the different fascicles of the LMM, which makes distinction of individual fascicles on imaging exceedingly challenging. For our review, this underlying anatomical complexity was further compounded by a lack of focused measurement methodologies or agreed muscle degeneration criteria used in the included studies. This resulted in a wide variety of approaches to investigate for associations between spinal pathology and paraspinal muscles changes, with outcomes that were difficult to compare or amalgamate for a more robust statistical analysis. In this regard, our findings were consistent with a recent narrative review by Kalichman et al. [62], and a proposed paraspinal muscle analysis methodology by Crawford et al. [63], each identifying a strong need to establish uniform methods for evaluating paraspinal muscle degeneration.

The limited quality assessment tool options for cross-sectional studies created a challenge, and while no generally accepted and valid tool was identified for looking at the associations between pathology and muscle changes, two options presented with the best potential: that developed by Downs and Black [28], and the Newcastle-Ottawa scale [64]. Although the Newcastle-Ottawa scale was designed specifically for observational studies, it was lacking in several reporting items we felt were important, was initially focussed on cohort and case control studies, and was still in the validation process. As both potential tools required modification, we determined to use a modified version of the Downs and Black risk of bias tool following the protocols of other similar published reviews [29, 30]. We also incorporated one additional modification by replacing a “clearly described intervention” item with a “clearly described assessment parameters” item, as we determined this to be an important and equivalent quality issue for our topic. These modifications may have had a small impact on overall risk of bias analysis, but this should have equally affected all studies. Varying degrees of familiarity with the tool between examiners may also have contributed to some of the initial non-agreement in the ROB analysis.

An insufficient number of studies were available to statistically assess for publication bias (e.g., funnel plots). However, while there is a potential for positive publication bias, the risk would seem fairly low in this review since the studies were observational and non-interventional, with no particular outcome from which those authors would benefit. Additionally, the moderate level of initial agreement for full text screening, and weak to moderate level of initial agreement for ROB analysis may have contributed to potential selection and/or quality bias; however, in the majority of cases the disagreement was either due to one examiner overlooking or misinterpreting a small inclusion/exclusion detail in a study, or related to complexities regarding how information was reported in relation to the ROB analysis criteria. In every case, full consensus was reached at the first meeting, with a relatively even mix of altered input between each examiner such that no one assessor dominated the review outcome.

The small number of studies included in each meta-analysis can reduce the precision of the estimate of the between-studies variance; the summary effect size results should be not be considered in isolation from the qualitative analysis.

Finally, this review does not address the issue of causality between pathology or altered muscle morphology and clinical findings. Additionally, in those instances where an association between spinal pathology and altered morphology was identified, no conclusions can be reached regarding the potential future clinical impact of these relationships.

## Recommendations

The high variability of approaches utilized to measure muscle morphology via imaging modalities created challenges for identifying any clear trends. In an attempt to promote some level of uniformity to muscle measurement techniques, in addition to and in conjunction with the protocols proposed by Crawford et al. [63], the following are recommended: 1) measurement ratios are preferable to standalone total or functional cross-sectional area measures, as they help to account for variations in individual patient anatomy and imaging parameters; 2) when calculating total cross-sectional area, measures should still include any central fat (i.e., measure to the vertebral arch boundaries for the multifidus), as this accounts for the total replacement of intra-epimysial muscle by fat; 3) when measuring functional muscle area, all obvious intramuscular fat should be excluded – this is potentially more time consuming, but provides a truer indication of functional muscle; 4) use of raw data from assessing muscle brightness (e.g., signal/density/echogenicity) is subject to variability between equipment and facilities – ratio differences in brightness may help overcome this limitation; 5) measurements should be analysed by individual spinal levels and specified in relation to the level of spinal pathology, as the data suggests this relationship to be of potential importance; 6) although measurements at any spinal level are acceptable, studies should at minimum include measures below the level of spinal pathology, particularly for disc herniations; 7) as an individual’s age, sex, and to a lesser degree BMI, all appear to have the potential to influence the morphology and/or appearance of the various paraspinal muscle groups, these three parameters should be clearly identified and accounted for during any analysis.

## Conclusions

Histologically, there was recurring evidence that fiber changes consistent with muscle denervation and re-innervation were associated with LDH when the uninvolved side muscles were used as the control. Insufficient biopsy evidence was available to analyse for relationships between arthrosis or stenosis and altered muscle fiber morphology. With imaging, the only relatively consistent finding was the apparent reduction in LMM functional muscle on the side of LDH and radiculopathy as symptoms became more chronic; however, several studies failed to separate acute from chronic patients in their analysis so the true differences relating to chronicity are unclear. Future studies should attempt to report and analyse chronic and acute patients separately to address this issue. No consistent imaging findings associated with LDH-related changes to the PMM were identified. Increased severity of facet arthrosis appeared to correlate with increased fatty infiltration of the PVM at the level of arthrosis. Any associations between spinal canal stenosis and altered muscle morphology were inconclusive.

Although a number of studies have looked at the potential impact of neurocompressive conditions on paraspinal muscle morphology, uncertainty remains – in large part due to the publication of a significant number of moderate to high risk of bias studies, and the variability of approaches used by these studies to assess for relationships. In patients with chronic radiculopathy, neurocompressive disorders seem to alter muscle morphology at or below the affected level. Future research should include more uniform methods and our proposed criteria may potentially improve the chance of determining if there are any clinically relevant associations between spinal pathology and muscle atrophy.

## Additional files

Additional file 1: Appendix 1. Database search parameters. (PDF 24 kb)

Additional file 2: Appendix 2. Systematic review selection/extraction form (PDF 24 kb)

Additional file 3: Appendix 3. Checklist for measuring risk of bias (PDF 39 kb)

Additional file 4: Appendix 4. Articles excluded at full-text review (PDF 66 kb)

Additional file 5: Extracted data and outcomes broken down by pathology and assessment methodology^†^ (PDF 174 kb)

## Abbreviations

CLBP: Chronic low back pain
CSA: Cross-sectional area
DDD: Degenerative disc disease
EMCL: Extramyocellular lipids
ESM: Erector spinae muscle
FCSA: Functional cross-sectional area
IMCL: Intramyocellular lipids
IS:US: Involved side : uninvolved side
LBP: Low back pain
LDH: Lumbar disc herniation
LMM: Lumbar multifidus muscle
MFI: Muscle-fat index
MLD: Muscle-laminar distance
MSF: Muscle strength factor
NR: Nerve root
PMM: Psoas major muscle
PVM: Paravertebral / Paraspinal muscle
QLM: Quadratus lumborum muscle
ROB: Risk of bias
SLR: Straight leg raise
SMD: Standardised mean difference
TCSA: Total cross-sectional area

## References

[1] Collaborators GDaIIaP. Global, regional, and national incidence, prevalence, and years lived with disability for 328 diseases and injuries for 195 countries, 1990-2016: A systematic analysis for the global burden of disease study 2016. Lancet. 2017;390(10100):1211-1259. 10.1016/S0140-6736(17)32154-2 PMC5605509 28919117

[2] Walker BF (2000). The prevalence of low back pain: A systematic review of the literature from 1966 to 1998. J Spinal Disord..

[3] Hoy D, Bain C, Williams G, March L, Brooks P, Blyth F (2012). A systematic review of the global prevalence of low back pain. Arthritis Rheum..

[4] Walker BF, Muller R, Grant WD (2003). Low back pain in Australian adults: The economic burden. Asia Pac J Public Health..

[5] Montgomery W, Sato M, Nagasaka Y, Vietri J (2017). The economic and humanistic costs of chronic lower back pain in Japan. ClinicoEconomics and Outcomes Research: CEOR..

[6] Koes B W, van Tulder M W, Thomas S (2006). Diagnosis and treatment of low back pain. BMJ.

[7] Lee S, Chan C, Lam T, Lam C, Lau N, Lau RW (2006). Relationship between low back pain and lumbar multifidus size at different postures. Spine (Phila Pa 1976)..

[8] Kjaer P, Bendix T, Sorensen JS, Korsholm L, Leboeuf-Yde C. Are MRI-defined fat infiltrations in the multifidus muscles associated with low back pain? BMC Med. 2007;5(2). 10.1186/1741-7015-5-2 PMC1796893 17254322

[9] Kamaz M, Kiresi D, Oguz H, Emlik D, Levendoglu F (2007). CT measurement of trunk muscle areas in patients with chronic low back pain. Diagn Interv Radiol..

[10] Hides J, Gilmore C, Stanton W, Bohlscheid E (2008). Multifidus size and symmetry among chronic LBP and healthy asymptomatic subjects. Man Ther..

[11] Wallwork TL, Stanton WR, Freke M, Hides JA (2009). The effect of chronic low back pain on size and contraction of the lumbar multifidus muscle. Man Ther..

[12] Fortin M, Macedo LG (2013). Multifidus and paraspinal muscle group cross-sectional areas of patients with low back pain and control patients: A systematic review with a focus on blinding. Phys Ther..

[13] Wong AYL, Parent EC, Funabashi M, Stanton TR, Kawchuk GN (2013). Do various baseline characteristics of transversus abdominis and lumbar multifidus predict clinical outcomes in nonspecific low back pain? A systematic review. Pain..

[14] Wong Arnold Y.L., Parent Eric C., Funabashi Martha, Kawchuk Gregory N. (2014). Do Changes in Transversus Abdominis and Lumbar Multifidus During Conservative Treatment Explain Changes in Clinical Outcomes Related to Nonspecific Low Back Pain? A Systematic Review. The Journal of Pain.

[15] Battie MC, Niemelainen R, Gibbons LE, Dhillon S (2012). Is level- and side-specific multifidus asymmetry a marker for lumbar disc pathology?. Spine Journal..

[16] Kim WH, Lee SH, Lee DY (2011). Changes in the cross-sectional area of multifidus and psoas in unilateral sciatica caused by lumbar disc herniation. Journal of Korean Neurosurgical Society..

[17] Chen YY, Pao JL, Liaw CK, Hsu WL, Yang RS (2014). Image changes of paraspinal muscles and clinical correlations in patients with unilateral lumbar spinal stenosis. Eur Spine J..

[18] Farshad M, Gerber C, Farshad-Amacker NA, Dietrich TJ, Laufer-Molnar V, Min K (2014). Asymmetry of the multifidus muscle in lumbar radicular nerve compression. Skeletal Radiol..

[19] Ploumis A, Michailidis N, Christodoulou P, Kalaitzoglou I, Gouvas G, Beris A (2011). Ipsilateral atrophy of paraspinal and psoas muscle in unilateral back pain patients with monosegmental degenerative disc disease. Br J Radiol..

[20] Yarjanian JA, Fetzer A, Yamakawa KS, Tong HC, Smuck M, Haig A (2013). Correlation of paraspinal atrophy and denervation in back pain and spinal stenosis relative to asymptomatic controls. PM and R..

[21] Kang CH, Shin MJ, Kim SM, Lee SH, Lee CS (2007). MRI of paraspinal muscles in lumbar degenerative kyphosis patients and control patients with chronic low back pain. Clin Radiol..

[22] Yoshihara K, Shirai Y, Nakayama Y, Uesaka S (2001). Histochemical changes in the multifidus muscle in patients with lumbar intervertebral disc herniation. Spine (Phila Pa 1976)..

[23] Dulor JP, Cambon B, Vigneron P, Reyne Y, Nougues J, Casteilla L (1998). Expression of specific white adipose tissue genes in denervation-induced skeletal muscle fatty degeneration. FEBS Lett..

[24] Carlson BM (2014). The biology of long-term denervated skeletal muscle. European Journal of Translational Myology..

[25] Steffens D, Hancock MJ, Maher CG, Williams C, Jensen TS, Latimer J (2014). Does magnetic resonance imaging predict future low back pain? A systematic review. Eur J Pain..

[26] Moher D, Liberati A, Tetzlaff J, Altman DG (2009). Preferred reporting items for systematic reviews and meta-analyses: The PRISMA statement. Ann Intern Med..

[27] Stroup DF, Berlin JA, Morton SC (2000). Meta-analysis of observational studies in epidemiology: A proposal for reporting. JAMA..

[28] Downs SH, Black N (1998). The feasibility of creating a checklist for the assessment of the methodological quality both of randomised and non-randomised studies of health care interventions. J Epidemiol Community Health..

[29] Mills K, Hunt MA, Leigh R, Ferber R (2013). A systematic review and meta-analysis of lower limb neuromuscular alterations associated with knee osteoarthritis during level walking. Clin Biomech (Bristol, Avon).

[30] Munn J, Sullivan SJ, Schneiders AG (2010). Evidence of sensorimotor deficits in functional ankle instability: A systematic review with meta-analysis. J Sci Med Sport..

[31] Kalichman L, Hodges P, Li L, Guermazi A, Hunter DJ (2010). Changes in paraspinal muscles and their association with low back pain and spinal degeneration: CT study. Eur Spine J..

[32] Kalichman L, Kim DH, Li L, Guermazi A, Hunter DJ (2010). Computed tomography-evaluated features of spinal degeneration: Prevalence, intercorrelation, and association with self-reported low back pain. The Spine Journal : Official Journal of the North American Spine Society..

[33] Yoshihara K, Nakayama Y, Fujii N, Aoki T, Ito H (2003). Atrophy of the multifidus muscle in patients with lumbar disk herniation: Histochemical and electromyographic study. Orthopedics..

[34] McHugh ML (2012). Interrater reliability: The kappa statistic. Biochemia Medica..

[35] Altinkaya Naime, Cekinmez Melih (2015). Lumbar multifidus muscle changes in unilateral lumbar disc herniation using magnetic resonance imaging. Skeletal Radiology.

[36] Boyacı A, Tutoğlu A, Boyacı N, Dokumacı DŞ (2015). MRI evaluation of fatty degeneration of paravertebral muscles in the patients with lumbar disc herniation with nerve root compression [Turkish]. Journal of Physical Medicine & Rehabilitation Sciences / Fiziksel Tup ve Rehabilitasyon Bilimleri Dergisi..

[37] Dangaria TR, Naesh O (1998). Changes in cross-sectional area of psoas major muscle in unilateral sciatica caused by disc herniation. Spine (Phila Pa 1976)..

[38] Hyun JK, Lee JY, Lee SJ, Jeon JY (2007). Asymmetric atrophy of multifidus muscle in patients with unilateral lumbosacral radiculopathy. Spine (Phila Pa 1976)..

[39] Kong BJ, Lim JS, Kim K (2014). A study on dispersion and rate of fat infiltration in the lumbar spine of patients with herniated nucleus polpusus. Journal of Physical Therapy Science..

[40] Bhadresha A, Lawrence OJ, McCarthy MJ (2016). A comparison of magnetic resonance imaging muscle fat content in the lumbar paraspinal muscles with patient-reported outcome measures in patients with lumbar degenerative disk disease and focal disk prolapse. Global Spine Journal..

[41] Fortin M, Lazary A, Varga PP, McCall I, Battie MC (2016). Paraspinal muscle asymmetry and fat infiltration in patients with symptomatic disc herniation. Eur Spine J..

[42] Frost LR, Brown SH (2016). Neuromuscular ultrasound imaging in low back pain patients with radiculopathy. Man Ther..

[43] Sun D, Liu P, Cheng J, Ma Z, Liu J, Qin T (2017). Correlation between intervertebral disc degeneration, paraspinal muscle atrophy, and lumbar facet joints degeneration in patients with lumbar disc herniation. Trials..

[44] Bajek S, Bobinac D, Bajek G, Vranić TS, Lah B, Dragojević DM (2000). Muscle fiber type distribution in multifidus muscle in cases of lumbar disc herniation. Acta Medica Okayama..

[45] Ford D, Bagnall KM, McFadden KD (1983). Analysis of vertebral muscle obtained during surgery for correction of a lumbar disc disorder. Acta Anat (Basel)..

[46] Mattila M, Hurme M, Alaranta H, Paljarvi L, Kalimo H, Falck B (1986). The multifidus muscle in patients with lumbar disc herniation. A histochemical and morphometric analysis of intraoperative biopsies. Spine (Phila Pa 1976)..

[47] Zhao WP, Kawaguchi Y, Matsui H, Kanamori M, Kimura T (2000). Histochemistry and morphology of the multifidus muscle in lumbar disc herniation - comparative study between diseased and normal sides. Spine (Phila Pa 1976)..

[48] Jowett RL, Fidler MW, Troup JD (1975). Histochemical changes in the multifidus in mechanical derangements of the spine. The Orthopedic Clinics of North America..

[49] Kalichman Leonid, Klindukhov Alexander, Li Ling, Linov Lina (2016). Indices of Paraspinal Muscles Degeneration. Clinical Spine Surgery.

[50] Sebro R, O'Brien L, Torriani M, Bredella MA (2016). Assessment of trunk muscle density using CT and its association with degenerative disc and facet joint disease of the lumbar spine. Skeletal Radiol..

[51] Yu B, Jiang K, Li X, Zhang J, Liu Z (2017). Correlation of the features of the lumbar multifidus muscle with facet joint osteoarthritis. Orthopedics..

[52] Abbas J, Slon V, May H, Peled N, Hershkovitz I, Hamoud K (2016). Paraspinal muscles density: A marker for degenerative lumbar spinal stenosis?. BMC Musculoskelet Disord..

[53] Jiang J, Wang H, Wang L, Zhang B, Guo Q, Yuan W (2017). Multifidus degeneration, a new risk factor for lumbar spinal stenosis: A case-control study. World Neurosurg..

[54] Ogon I, Takebayashi T, Takashima H, Morita T, Yoshimoto M, Terashima Y (2017). Magnetic resonance spectroscopic analysis of multifidus muscles lipid content and association with spinopelvic malalignment in chronic low back pain. Br J Radiol..

[55] Mannion AF, Dumas GA, Cooper RG, Espinosa FJ, Faris MW, Stevenson JM (1997). Muscle fibre size and type distribution in thoracic and lumbar regions of erector spinae in healthy subjects without low back pain: Normal values and sex differences. J Anat..

[56] Kalichman L, Li L, Kim DH, Guermazi A, Berkin V, O'Donnell CJ (2008). Facet joint osteoarthritis and low back pain in the community-based population. Spine (Phila Pa 1976)..

[57] Kottlors M, Glocker FX (2008). Polysegmental innervation of the medial paraspinal lumbar muscles. Eur Spine J..

[58] Hodges PW, James G, Blomster L, Hall L, Schmid A, Shu C (2015). Multifidus muscle changes after back injury are characterized by structural remodeling of muscle, adipose and connective tissue, but not muscle atrophy: Molecular and morphological evidence. Spine (Phila Pa 1976)..

[59] Lonnemann ME, Paris SV, Gorniak GC (2008). A morphological comparison of the human lumbar multifidus by chemical dissection. J Man Manip Ther..

[60] Cornwall J, Stringer MD, Duxson M (2011). Functional morphology of the thoracolumbar transversospinal muscles. Spine (Phila Pa 1976)..

[61] Macintosh JE, Valencia F, Bogduk N, Munro RR (1986). The morphology of the human lumbar multifidus. Clin Biomech (Bristol, Avon).

[62] Kalichman Leonid, Carmeli Eli, Been Ella (2017). The Association between Imaging Parameters of the Paraspinal Muscles, Spinal Degeneration, and Low Back Pain. BioMed Research International.

[63] Crawford RJ, Cornwall J, Abbott R, Elliott JM (2017). Manually defining regions of interest when quantifying paravertebral muscles fatty infiltration from axial magnetic resonance imaging: A proposed method for the lumbar spine with anatomical cross-reference. BMC Musculoskelet Disord..

[64] Wells G, Shea B, O'Connell D, Peterson J (2014). Welch V et al. The Newcastle-Ottawa Scale (NOS) for assessing the quality of nonrandomised studies in meta-analyses Ottawa Hospital Research Institute.

