# Supplementary material for: Relationships between paraspinal muscle morphology and neurocompressive conditions of the lumbar spine: a systematic review with meta-analysis
Source: BMC Musculoskelet Disord. 2018 Sep 27;19:351. doi: 10.1186/s12891-018-2266-5 (PMC6161433; doi:10.1186/s12891-018-2266-5)
Supplement: Supplementary file 1 — Appendix 1. Database search parameters. (PDF 24 kb) [file 12891_2018_2266_MOESM1_ESM.pdf]

Appendix 1 – Database search parameters

Embase search protocol:

1. (trunk or lumbar or lumbo* or abdomen or core or ,low back, or ,lower back, or sacrum or sacral).mp
2. exp muscle/
3. (muscular or neuromuscular or multifid* or ,erector spinae, or paraspinal* or paravertebral or ,iliocostalis lumborum,).mp
4. 2 or 3
5. (muscle and (fiber* or fibre* or fascicle* or morphometry or asymmetry or size or morphology or architecture or hypertrophy or atrophy or wasting or hyperplasia or circumference or volume or diameter or thickness)) or (muscular and (fiber* or fibre* or fascicle* or morphometry or asymmetry or size or morphology or architecture or hypertrophy or atrophy or wasting or hyperplasia or circumference or volume or diameter or thickness)) or ,cross sectional, or ,cross-sectional, or ,fat replacement, or ,fatty replacement, or ,intramuscular fat, or ,intramuscular fatty tissue, or ,functional CSA, or ,functional cross sectional area, or ,functional cross-sectional area,).mp
6. (degenerat* or ,intervertebral disc degeneration, or ,intervertebral disk degeneration, or ,intervertebral disc displacement, or ,intervertebral disk displacement, or ,degenerative disc disease, or ,degenerative disk disease, or ,degenerative spinal disease, or Spondylosis or ,Spinal Stenosis, or ,disc bulg*, or ,disc protrusion*, or ,disc extrusion*, or ,disk bulg*, or ,disk protrusion*, or ,disk extrusion*, or (facet and (degeneration or arthrosis or osteoarthrosis or osteoarthritis or arthropathy)) or (apophyseal and (degeneration or arthrosis or osteoarthrosis or osteoarthritis or arthropathy)) or ,modic change, or ,modic changes, or ,lateral recess stenosis, or ,foraminal stenosis, or ,central canal stenosis, or ,spinal fracture*, or ,pars fracture*, or ,pars defect*, or spondylolysis or spondylolisthesis or alignment or postur* or ,postural alignment, or swayback or ,sway-back, or ,sway back, or ,lumbar kyphosis, or ,lumbar Scheuermann*, or ,spinal curv*, or scoliosis).mp
7. (Radiculopathy or Sciatica or ,referred pain, or paresthesia or ,leg pain, or ,lower limb pain, or ,lower extremity pain, or ,leg weakness, or ,lower limb weakness, or ,lower extremity weakness, or claudication or ,neurogenic claudication, or ,radicular pain, or ,discogenic pain, or ,nerve compression, or ,low back pain, or ,lower back pain, or ,lumbar pain, or lumbago or lumbalgia or LBP).mp
8. 6 or 7
9. 1 and 4 and 5 and 8
10. (,magnetic resonance imaging, or MRI or ultrasonography or ,diagnostic ultrasound, or DUS or ,X-ray computed tomography, or ,computed tomography, or ,computer* tomography, or CT).mp
11. (cadaver* or biopsy or immunohistochem* or immunohistolog* or histolog*).mp
12. 10 or 11
13. 9 and 12

Medline search protocol (used for PubMed, Web of Science, SPORTDiscus, Cinahl):

S1 (Region)*:* trunk OR lumbar OR lumbo* OR abdomen OR core OR "low back" OR "lower back" OR sacrum OR sacral *(All fields +MeSH)*

S2: muscle *(MeSH)*

S3 (Muscle type)*:* muscular OR neuromuscular OR multifid* OR "erector spinae" OR paraspinal* OR paravertebral OR "iliocostalis lumborum" *(All fields + MeSH)*

S4: S2 OR S3

*S5 (*Muscle Morphology)*:* "cross sectional" OR "cross-sectional" OR “fat replacement” OR “fatty replacement” OR “intramuscular fat” OR “intramuscular fatty tissue” OR “intramuscular adipose” OR “intramuscular adipose tissue” OR “functional CSA” OR “functional cross sectional area” OR “functional cross-sectional area” OR (muscle AND (hypertrophy OR asymmetry OR size OR morphology OR architecture OR hyperplasia OR circumference OR volume OR diameter OR thickness OR atrophy OR wasting OR morphometry OR fiber OR fibers OR fibre OR fibres OR fascicle OR fascicles)) OR (muscular AND (hypertrophy OR asymmetry OR size OR morphology OR architecture OR hyperplasia OR circumference OR volume OR diameter OR thickness OR atrophy OR wasting OR morphometry OR fiber OR fibers OR fibre OR fibres OR fascicle OR fascicles)) *(All fields +MeSH)*

*S6 (*Pathology and posture)*:* degenerat* OR “intervertebral disc degeneration” OR "intervertebral disk degeneration" OR "degenerative disc disease" OR "degenerative disk disease" OR “degenerative spinal disease” OR spondylosis OR "intervertebral disc displacement” OR "intervertebral disk displacement” OR (disc AND (bulge OR bulges OR herniation OR herniations OR protrusion OR protrusions OR prolapse OR prolapses OR extrusion OR extrusions OR sequest*)) OR (disk AND (bulge OR bulges OR herniation OR herniations OR protrusion OR protrusions OR prolapse OR prolapses OR extrusion OR extrusions OR sequest*)) OR (facet AND (degeneration OR arthrosis OR osteoarthrosis OR osteoarthritis OR arthropathy)) OR (apophyseal AND (degeneration OR arthrosis OR osteoarthrosis OR osteoarthritis OR arthropathy)) OR "modic change” OR "modic changes" OR “spinal stenosis" OR "lateral recess stenosis" OR "foraminal stenosis" OR "central canal stenosis" OR “spinal fracture" OR "spinal fractures" OR "pars fracture" OR "pars fractures" OR "pars defect" OR "pars defects" OR spondylolysis OR spondylolisthesis OR "spinal curv*" OR alignment OR postur* OR swayback OR “sway-back” OR “sway back” OR “lumbar kyphosis” OR “lumbar Scheuermann*” OR scoliosis *(All fields +MeSH)*

S7 (Clinical symptoms)*:* radiculopathy OR sciatica OR “referred pain" OR “discogenic pain” OR paresthesia OR claudication OR "neurogenic claudication" OR "radicular pain" OR (pain AND (leg OR "lower limb" OR "lower extremity" OR “low back” OR “lower back” OR lumbar)) OR (weakness AND (leg OR "lower limb" OR "lower extremity")) OR “nerve compression” OR lumbago OR lumbalgia OR LBP *(All fields +MeSH)*

S8: S6 OR S7

S9: S1 AND S4 AND S5 AND S8

S10 (Imaging method)*:* “magnetic resonance imaging” OR “nuclear magnetic resonance imaging” OR MRI OR “MR imaging” OR ultrasonography OR “diagnostic ultrasound” OR DUS OR “X-ray computed tomography” OR “computed tomography” OR “computer* tomography” OR CT OR “CAT scan” *(All fields +MeSH)*

S11: cadaver* OR biopsy OR immunohistochem* OR immunohistolog* OR histolog* *(All fields +MeSH)*

S12: S10 OR S11

S13: S9 AND S12

PEDro search protocol:

Body Part: lumbar spine, sacroiliac joint or pelvis AND (**“**magnetic resonance imaging” OR MRI OR “MR imaging” OR ultrasonography OR “diagnostic ultrasound” OR DUS OR “X-ray computed tomography” OR “computed tomography” OR “computer* tomography” OR CT OR “CAT scan” OR cadaver* OR biopsy OR immunohistochem* OR immunohistolog* OR histolog*)

Body Part: lumbar spine, sacroiliac joint or pelvis AND (neuromuscular OR multifid* OR "erector spinae" OR “paraspinal muscle” OR “paravertebral muscle” OR "iliocostalis lumborum")

Body Part: lumbar spine, sacroiliac joint or pelvis AND muscle AND ("cross sectional" OR "cross-sectional" OR “fat replacement” OR “fatty replacement” OR “intramuscular fat” OR “intramuscular fatty tissue” OR “intramuscular adipose” OR “intramuscular adipose tissue” OR “functional CSA” OR “functional cross sectional area” OR “functional cross-sectional area” OR hypertrophy OR asymmetry OR size OR morphology OR architecture OR hyperplasia OR circumference OR volume OR diameter OR thickness OR atrophy OR wasting OR morphometry OR fiber OR fibers OR fibre OR fibres OR fascicle OR fascicles)

Body Part: lumbar spine, sacroiliac joint or pelvis AND (degenerat* OR “intervertebral disc degeneration” OR "intervertebral disk degeneration" OR "degenerative disc disease" OR "degenerative disk disease" OR “degenerative spinal disease” OR spondylosis OR "intervertebral disc displacement” OR "intervertebral disk displacement” OR (disc AND (bulge OR bulges OR herniation OR herniations OR protrusion OR protrusions OR prolapse OR prolapses OR extrusion OR extrusions OR sequestration or sequestrations)) OR (“facet joint” AND (degeneration OR arthrosis OR osteoarthrosis OR osteoarthritis OR arthropathy)) OR (“apophyseal joint” AND (degeneration OR arthrosis OR osteoarthrosis OR osteoarthritis OR arthropathy)) OR "modic change” OR "modic changes" OR “spinal stenosis" OR "lateral recess stenosis" OR "foraminal stenosis" OR "central canal stenosis" OR “spinal fracture" OR "spinal fractures" OR "pars fracture" OR "pars fractures" OR "pars defect" OR "pars defects" OR spondylolysis OR spondylolisthesis OR "spinal curve" OR “spinal curves” OR “spinal curvature” OR “spinal curvatures” OR alignment OR postur* OR swayback OR “sway-back” OR “sway back” OR “lumbar kyphosis” OR “lumbar Scheuermann*” OR scoliosis)

Body Part: lumbar spine, sacroiliac joint or pelvis AND *Problem*: pain AND (radiculopathy OR sciatica OR referred OR discogenic OR paresthesia OR claudication OR "neurogenic claudication" OR radicular OR “leg pain” OR "lower limb pain" OR "lower extremity pain" OR “nerve compression”)

Body Part: lumbar spine, sacroiliac joint or pelvis AND *Problem*: muscle weakness AND (leg OR "lower limb" OR "lower extremity")
