# Supplementary material for: Relationships between paraspinal muscle morphology and neurocompressive conditions of the lumbar spine: a systematic review with meta-analysis
Source: BMC Musculoskelet Disord. 2018 Sep 27;19:351. doi: 10.1186/s12891-018-2266-5 (PMC6161433; doi:10.1186/s12891-018-2266-5)
Supplement: Supplementary file 2 — Appendix 2. Systematic review selection/extraction form (PDF 24 kb) [file 12891_2018_2266_MOESM2_ESM.pdf]

Appendix 2: Systematic review selection / extraction form

Articles Included After Initial Title/Abstract Review

- First author (Surname, Initials):
- Second author:
- Title of article:
- Source details (eg, Journal name and details, PhD thesis source):

Final Inclusion Details (Full Text Screening):

This research in this article was:

- Human-based: Yes / No
- Based on an observational study, or on a surgical or clinical trial [with no history of prior back surgery]: Yes / No
    - o If YES, indicate if: Observational (O); Surgical trial (S); Clinical trial (C):
- Inclusive of a control group for paraspinal ms comparison: Yes / No
    - o If a surgical or clinical trial, comparison with control must have been "pre-interventional" WITH analysis of the pre-interventional comparison groups. If not, input NO]:
    - o If YES, was the comparison to a "normal control" (N) or an "abnormal control" (A)?
- Assessing the morphology/histology of muscles in the lumbosacral region ie, LM, EX, QL, Psoas, or “paraspinal”): Yes / No
    - o If only looking at function or changes to morphology following a functional action (eg, flexion), input NO]
- Assessing pathologic or clinical conditions localized to the lumbar region and/or lower extremities, AND inferentially correlating to muscle findings (but NOT primary muscle diseases that could also affect areas beyond the lumbar region/L.E.s): Yes / No
    - o Caveat: if assessing scoliosis in the thoracic AND lumbar regions, input YES]
- If you answered NO to any of the above, note your specific reason(s) OR indicate any specific details to highlight for included studies:
    - o Reason(s):
- If you answered YES to all of the above, input Y here and proceed to Data Extraction. If you answered NO to any of the above, Exclude the article (input N here).

Data Extraction Details:

- What was the study aim/objective?
- What was the study design (e.g., case control, cohort, cross-sectional, pre-surgical, clinical trial, etc.)?
- What were the defined characteristics of *primary participants* (e.g., inclusion/exclusion criteria, nationality, pop. source, etc.)?
- What was the primary subject sample size? If no sample size indicate, input "0"

    - If provided, what was the source population size (or, % of sample from source population)?
    - If provided, what was the recruitment population size (or, % of sample from recruited population)?
- What was the sampling method (e.g., random, convenience, consecutive)?
- Note the distribution of principle confounders [Age, sex, BMI. Refer to line 5 in ROB section for additional confounders]:
- What were the defined characteristics of the *control/comparison group* (e.g., normal, hospital-based, age-matched, etc.) AND Criteria (e.g., no back pain, acute back pain, no [type of pathology], own control - unaffected side or level, etc.)?
    - If subjects not own control, what was comparison group sample size?
- What were the defined characteristics of *any other controls* (e.g., normal, hospital-based, age-matched, etc.) AND Criteria (e.g., no back pain, acute back pain, no [type of pathology], own control - unaffected side or level, etc.)?
    - What was additional control/comparison sample size?
- If applicable, note the distribution of principle confounders in controls [Age, sex, BMI. Refer to line 5 in ROB section for additional confounders]:
- If present, highlight any differences in recruitment between groups [This could include differences in the source population (e.g., hospital vs general population) or the timing of recruitment (e.g., at different years). If none, type "None" above.]:
- Indicate any blinding procedures that were used. Also note any issues related to blinding (e.g., measurements taken at same image slice as disc herniation):
- Indicate which modality/process was used to assess the muscles (e.g., MRI, CT, DUS, biopsy, etc.):
- Define the PATHOLOGY variables or the CLINICAL variables assessed (e.g., disc herniation or degeneration, facet joint degeneration, spinal canal stenosis, spondylolisthesis, scoliosis, lumbar kyphosis, sway back, low back pain, radiculopathy, leg weakness, atrophy, paresthesia, etc.):
- Indicate which muscle(s) were assessed (e.g., multifidus, erector spinae, paraspinal muscle, etc.):
- Indicate which muscle characteristics were assessed (e.g., atrophy, fatty infiltration, functional muscle, muscle cell type, etc.), and what measurement variables were used to assess the muscles (e.g., cross-sectional muscle / fat, total cross-sectional muscle, semi-quantitative muscle grading, histochemical analysis, etc.):

Provide the study outcomes concerning the strength of relationship /association between muscle and assessed variables:

- Outcomes 1 up to 7:
    - Outcomes (Key stats*) [* Key stats could include actual scores, P values, SE, SD, CIs, etc. Provide a brief summary, but list values exactly as recorded in article. If data is too extensive, note the location of the data within the document].
- Were any of the above outcomes based on "data dredging" (If yes, indicate which outcome(s); if no, type "none")?
