# Supplementary material for: Relationships between paraspinal muscle morphology and neurocompressive conditions of the lumbar spine: a systematic review with meta-analysis
Source: BMC Musculoskelet Disord. 2018 Sep 27;19:351. doi: 10.1186/s12891-018-2266-5 (PMC6161433; doi:10.1186/s12891-018-2266-5)
Supplement: Supplementary file 3 — Appendix 3. Checklist for measuring risk of bias (PDF 39 kb) [file 12891_2018_2266_MOESM3_ESM.pdf]

Appendix 3: Checklist for measuring risk of bias

Reporting

1. Is the hypothesis/aim/objective of the study clearly described?

○ Yes (1) ○ No (0)

2. Are the main outcomes to be measured clearly described in the Introduction or Methods section? *For example, which muscles or muscle changes were assessed?) If the main outcomes are first mentioned in the Results section, the question should be answered No.*

○ Yes (1) ○ No (0)

3. Are the characteristics of the patients included in the study clearly described? *In cohort studies and trials, inclusion and/or exclusion criteria should be given. In case-control studies, a case-definition and the source for control should be given.*

○ Yes (1) ○ No (0)

4. Are the principal assessment parameters in the study clearly described? *Assessment parameters include: 1) type / subtype of imaging used; 2) how muscle changes were measured; 3) type of control/comparison used.*

○ Yes (2) ○ Partially (1) ○ No (0)

5. Are the distributions of principal confounders in each group of subjects to be compared clearly described? *Principal confounders include: 1) type, severity, or chronicity of pathology or clinical findings (if not compared to a normal control); 2) non-assessed low back co-morbidities (if any); 3) subject demographics; 4) subject body type / size.*

○ Yes (2) ○ Partially (1) ○ No (0)

6. Are the main findings of the study clearly described? *Simple outcome data (including denominators and numerators) should be reported for all major findings so that the reader can check the major analyses and conclusions. (This question does not cover statistical tests which are considered below).*

○ Yes (1) ○ No (0)

7. Does the study provide estimates of the random variability in the data for the main outcomes? *In non-normally distributed data the inter-quartile range of results should be reported. In normally distributed data the standard error, standard deviation or confidence intervals should be reported. If the distribution of the data is not described, it must be assumed that the estimates used were appropriate and the question should be answered Yes.*

○ Yes (1) ○ No (0)

10. Have actual probability values been reported? *(e.g. 0.035 rather than <0.05 for the main outcomes except where the probability value is less than 0.001.)*

○ Yes (1) ○ No (0)

External validity

All the following criteria attempt to address the representativeness of the findings of the study and whether they may be generalised to the population from which the study subjects were derived.

11. Were the subjects asked to participate in the study representative of the entire population from which they were recruited? *The study must identify the source population for patients and describe how the patients were selected. Patients would be representative if they comprised the entire source population, an unselected sample of consecutive patients, or a random sample. Random sampling is only feasible where a list of all members of the relevant population exists. Where a study does not report the proportion of the source population from which the patients are derived, the question should be answered as Unable to determine.*

○ Yes (1) ○ No (0) ○ Unable to determine (0)

12. Were those subjects who were prepared to participate representative of the entire population from which they were recruited? *The proportion of those asked who agreed should be stated. Validation that the sample was representative would include demonstrating that the distribution of the main confounding factors was the same in the study sample and the source population.*

○ Yes (1) ○ No (0) ○ Unable to determine (0)

Internal validity - bias

15. Was an attempt made to blind those measuring the main outcomes of the intervention? *For the purpose of this study, the "intervention" can include the underlying condition or the imaging modality (e.g., blinded to the underlying pathology or complaint, or to other's measurements).*

○ Yes (1) ○ No (0) ○ Unable to determine (0)

16. If any of the results of the study were based on "data dredging", was this made clear? *Any analyses that had not been planned at the outset of the study should be clearly indicated. If no retrospective unplanned subgroup analyses were reported, then answer Yes.*

○ Yes (1) ○ No (0) ○ Unable to determine (0)

18. Were the statistical tests used to assess the main outcomes appropriate? *The statistical techniques used must be appropriate to the data. For example nonparametric methods should be used for small sample sizes. Where little statistical analysis has been undertaken but where there is no evidence of bias, the question should be answered Yes. If the distribution of the data (normal or not) is not described it must be assumed that the estimates used were appropriate and the question should be answered Yes.*

○ Yes (1) ○ No (0) ○ Unable to determine (0)

20. Were the main outcome measures used accurate (valid and reliable)? *For studies where the outcome measures are clearly described, the question should be answered Yes. For studies which refer to other work or that demonstrates the outcome measures are accurate, the question should be answered as Yes.*

○ Yes (1) ○ No (0) ○ Unable to determine (0)

Internal validity - confounding (selection bias)

21. Were the patients in different intervention groups (trials and cohort studies) or were the cases and controls (case-control / cross-sectional studies) recruited from the same population? *For example, patients for all comparison groups should be selected from the same hospital. The question should be answered Unable to determine for cohort, case-control, and cross-sectional studies where there is no information concerning the source of patients included in the study.*

○ Yes (1) ○ No (0) ○ Unable to determine (0)

22. Were study subjects in different intervention groups (trials and cohort studies) or were the cases and controls (case-control / cross-sectional studies) recruited over the same period of time? *For a study which does not specify the time period over which patients were recruited, the question should be answered as Unable to determine.*

○ Yes (1) ○ No (0) ○ Unable to determine (0)

25. Was there adequate adjustment for confounding in the analyses from which the main findings were drawn? *This question should be answered no* <u>*for trials*</u> *if: the main conclusions of the study were based on analyses of treatment rather than intention to treat; the distribution of known confounders in the different treatment groups was not described; or the distribution of known confounders differed between the treatment groups but was not taken into account in the analyses.* <u>*In non-randomised studies*</u> *if the effect of the main confounders was not investigated or confounding was demonstrated but no adjustment was made in the final analyses the question should be answered as No.*

○ Yes (1) ○ No (0) ○ Unable to determine (0)

Note: The following modifications were made to the original ROB tool published by Downs and Black:

A) Item 2: examples related to this study content have been included for clarity
B) Item 4: the original item asked if the interventions of interest were clearly described. This item related to treatment interventions and as such was not germane to our review. It was revised to include an assessment parameter not addressed by the other items.
C) Item 5. A list of principal confounders relevant to our review was included for clarity.
D) Item 8 (adverse events from intervention), item 9 (patients lost to follow-up), item 13 (details related to location of treatment) and item 14 (blinding to treatment intervention) were removed as they were not germane to our review topic.
E) Item 15: Definition of "intervention" added, which allowed for inclusion of measurements of non-treatment related outcomes.
F) Item 17 (intervention follow-up time) and item 19 (intervention compliance) were removed as they were not germane to our review topic.
G) Items 21 and 22: For assessor clarity, "cross-sectional" was included in the study types.
H) Item 23 (intervention randomisation), item 24 (randomisation concealment), item 26 (patient loss to follow-up), and item 27 (power – clinically important intervention effect) were removed as they were not germane to our review topic.

NB: the addition of topic-specific explanatory notes, and the removal of treatment-specific items from the original ROB tool, was done in a manner consistent with other published modifications of this tool for observational studies.
