# Supplementary material for: Relationships between paraspinal muscle morphology and neurocompressive conditions of the lumbar spine: a systematic review with meta-analysis
Source: BMC Musculoskelet Disord. 2018 Sep 27;19:351. doi: 10.1186/s12891-018-2266-5 (PMC6161433; doi:10.1186/s12891-018-2266-5)
Supplement: Supplementary file 4 — Appendix 4. Articles excluded at full-text review (PDF 66 kb) [file 12891_2018_2266_MOESM4_ESM.pdf]

Appendix 4 – Articles excluded at full-text review

Abdelrahman H, Nahleh KA, et al. Psoas muscle changes associated with primary degenerative lumbar scoliosis. Eur Spine J. 2017;23:S580.

Akbari A, Khorashadizadeh S, Abdi G. The effect of motor control exercise versus general exercise on lumbar local stabilizing muscles thickness: randomized controlled trial of patients with chronic low back pain. Journal of Back and Musculoskeletal Rehabilitation 2008;21(2):105-112.

Alaranta H, Tallroth K, et al. Fat content of lumbar extensor muscles and low back disability: A radiographic and clinical comparison. Journal of Spinal Disorders 1993 6(2): 137-140.

Amabile C, Moal B, et al. Estimation of spinopelvic muscles' volumes in young asymptomatic subjects: a quantitative analysis. Surg Radiol Anat. 2017;39(4):393-403.

Anderson DE, Quinn E, et al. Associations of Computed Tomography-Based Trunk Muscle Size and Density With Balance and Falls in Older Adults. Gerontol A Biol Sci Med Sci. 2016;71(6):811-6.

Andersen M, Lee D, et al. Morphology of the lumbar multifidus in persons with back pain: a quantitative and qualitative analysis. J Orthop Phys Ther 2005;35(1):A13-A.

Antony J, McGuinness K, et al. An interactive segmentation tool for quantifying fat in lumbar muscles using axial lumbar-spine MRI. Irbm. 2016;37(1):11-22.

Arshad R, Zander T, et al. Influence of lumbar spine rhythms and intra-abdominal pressure on spinal loads and trunk muscle forces during upper body inclination. Med Eng Phys. 2016;38(4):333-8.

Arts M, Brand R, et al. Does minimally invasive lumbar disc surgery result in less muscle injury than conventional surgery? A randomized controlled trial. European Spine Journal 2011 20(1): 51-57.

Bardach C, Friedrich K, et al. Is there a correlation between the degree of fatty degeneration of dorsal paraspinal muscles and degenerative abnormalities of the lumbar spine? Skeletal Radiol. 2013;42 (6):878.

Banno T, Yamato Y, et al. Assessment of the Cross-Sectional Areas of the Psoas Major and Multifidus Muscles in Patients With Adult Spinal Deformity: A Case-Control Study. Clinical spine surgery. 2017;30(7):E968-e73.

Barrios C, Maruenda A, et al. MRI assessment of fat infiltration and atrophy of the lumbar muscles after one single level fusion with topping-off dynamic stabilization. Eur Spine J. 2014;23:S579-S80.

Belavy DL, Armbrecht G, et al. Countermeasures against lumbar spine deconditioning in prolonged bed rest: Resistive exercise with and without whole body vibration. Journal of Applied Physiology 2010;109(6):1801-1811.

Belavy DL, Armbrecht G, et al. Muscle atrophy and changes in spinal morphology: Is the lumbar spine vulnerable after prolonged bed-rest? Spine (Phila Pa 1976) 2011;36(2): 137-145.

Belavy DL, Bansmann PM, et al. Changes in intervertebral disc morphology persist 5 mo after 21-day bed rest. Journal of Applied Physiology 2011;111(5): 1304-1314.

Beneck GJ. Multifidus morphology, fatigability and activation in persons with chronic unilateral low back pain. PhD thesis, USC 2010.

Berglund L, Aasa B, et al. Effects of Low-Load Motor Control Exercises and a High-Load Lifting Exercise on Lumbar Multifidus Thickness: A Randomized Controlled Trial. Spine (Phila Pa 1976). 2017;42(15):E876-e82.

Bishop MD, Horn ME, et al. Magnitude of spinal muscle damage is not statistically associated with exercise-induced low back pain intensity. Spine J 2011;11(12): 1135-1142.

Boissiere L, Moal B, et al. Lumbar spinal muscles and spinal canal study by MRI three-dimensional reconstruction in adult lumbar spinal stenosis. Orthop Traumatol Surg Res. 2017 DOI: 10.1016/j.otsr.2016.10.025.

Bok DH, Kim J, Kim TH. Comparison of MRI-defined back muscles volume between patients with ankylosing spondylitis and control patients with chronic back pain: age and spinopelvic alignment matched study. Eur Spine J. 2017;26(2):528-37

Bouche KG, Vanovermeire O, et al. Computed tomographic analysis of the quality of trunk muscles in asymptomatic and symptomatic lumbar discectomy patients. BMC Musculoskelet Disord 2011;12: 65.

Bresnahan LE, Smith JS, et al. Assessment of Paraspinal Muscle Cross-sectional Area After Lumbar Decompression: Minimally Invasive Versus Open Approaches. Clinical spine surgery. 2017;30(3):E162-E8.

Bruno AG, Mokhtarzadeh H, et al. Incorporation of CT-based measurements of trunk anatomy into subject-specific musculoskeletal models of the spine influences vertebral loading predictions. J Orthop Res. 2017;35(10):2164-73.

Bruno AG, Mokhtarzadeh H. Incorporation of CT-based measurements of trunk anatomy into subject-specific musculoskeletal models of the spine influences vertebral loading predictions. J Orthop Res. 2017 DOI: 10.1002/jor.23524.

Burgstaller JM, Schuffler PJ, et al. Is There an Association Between Pain and Magnetic Resonance Imaging Parameters in Patients With Lumbar Spinal Stenosis? Spine (Phila Pa 1976) 2016;41(17):E1053-62.

Burkhart KA, Bruno AG, et al. Estimating apparent maximum muscle stress of trunk extensor muscles in older adults using subject-specific musculoskeletal models. J Orthop Res. 2017 DOI: 10.1002/jor.23630.

Castillo ER, Hsu C, et al. Testing biomechanical models of human lumbar lordosis variability. Am J Phys Anthropol. 2017 10.1002/ajpa.23189.

Chagas JCM, Schmidt B, et al. Histochemical study of lumbar rotator muscles in patients with adolescent idiopathic scoliosis. [Portuguese] Revista Brasileira de Ortopedia 1998;33(2): 111-118.

Chang DG, Healey RM, et al. Lumbar Spine Paraspinal Muscle and Intervertebral Disc Height Changes in Astronauts After Long-Duration Spaceflight on the International Space Station. Spine (Phila Pa 1976). 2016;41(24):1917-24.

Chen XQ, Chen JT, et al. Expressions of desmin and vimentin in the paraspinal muscles of patients with idiopathic scoliosis. [Chinese] Journal of Clinical Rehabilitative Tissue Engineering Research 2007;11(19): 3694-3697.

Chen YY, Pao JL, et al. Image changes of paraspinal muscles and clinical correlations in patients with unilateral lumbar spinal stenosis. European Spine Journal 2014;23(5): 999-1006.

Chiu JC. Morphological studies on the erector spinae muscle in sixty consecutive scoliotic patients. Nippon Seikeigeka Gakkai zasshi 1988;62(12): 1163-1175.

Choi MK, Kim SB. Do Trunk Muscles Affect the Lumbar Interbody Fusion Rate?: Correlation of Trunk Muscle Cross Sectional Area and Fusion Rates after Posterior Lumbar Interbody Fusion Using Stand-Alone Cage. J Korean Neurosurg Soc. 2016;59(3):276-81.

Choi MK, Kim SB, et al. Cross-Sectional Area of the Lumbar Spine Trunk Muscle and Posterior Lumbar Interbody Fusion Rate: A Retrospective Study. Clinical spine surgery. 2016 10.1097/bsd.0000000000000424.

Chung S, Lee J, Yoon J. Effects of Stabilization Exercise Using a Ball on Multifidus Cross-Sectional Area in Patients with Chronic Low Back Pain. Journal of Sports Science and Medicine 2013;12(3): 533-541.

Clark B, Walkowski S, et al. Muscle functional magnetic resonance imaging and acute low back pain: a pilot study to characterize lumbar muscle activity asymmetries and examine the effects of osteopathic manipulative treatment. Osteopath Med Prim Care 2009;3:7.

Cooper RG, Freemont AJ, et al. Paraspinal muscle fibrosis: a specific pathological component in ankylosing spondylitis. Ann Rheum Dis 1991;50(11): 755-759.

Cooper RG, St Clair Forbes W, Jayson MI. Radiographic demonstration of paraspinal muscle wasting in patients with chronic low back pain. Br J Rheumatol 1992;31(6): 389-394.

Crawford RJ, Cornwall J, et al. Manually defining regions of interest when quantifying paravertebral muscles fatty infiltration from axial MRI: a proposed method for the lumbar spine with anatomical cross-reference. BMC Musculoskelet Disord. 2017;18(1):25.

Crawford RJ, Filli L, et al. Age- and Level-Dependence of Fatty Infiltration in Lumbar Paravertebral Muscles of Healthy Volunteers. AJNR American journal of neuroradiology. 2016;37(4):742-8.

Crawford RJ, Volken T, et al. Rate of lumbar paravertebral muscle fat infiltration versus spinal degeneration in asymptomatic populations: an age-aggregated cross-sectional simulation study. Scoliosis and spinal disorders. 2016;11:21.

Crewe H, Campbell A, et al. Lumbo-Pelvic Biomechanics and Quadratus Lumborum Asymmetry in Cricket Fast Bowlers. Medicine & Science in Sports & Exercise 2013;45(4): 778-783.

Crossman K, Mahon M, et al. Chronic low back pain-associated paraspinal muscle dysfunction is not the result of a constitutionally determined "adverse" fiber-type composition. Spine (Phila Pa 1976) 2004;29(6): 628-634.

Cuesta-Vargas A, Gonzalez-Sanchez M. Ability to discriminate between healthy and low back pain sufferers using ultrasound during maximum lumbar extension. Arch Phys Med Rehabil 2014;95(6): 1093-1099.

Dahlqvist JR, Vissing CR, et al. Fat Replacement of Paraspinal Muscles with Aging in Healthy Adults. Med Sci Sports Exerc. 2017;49(3):595-601.

Danneels LA, Cools AM, et al. The effects of three different training modalities on the cross-sectional area of the paravertebral muscles. Scandinavian Journal of Medicine & Science in Sports 2001;11(6):335-341.

Danneels LA, Vanderstraeten GG, et al. Effects of three different training modalities on the cross sectional area of the lumbar multifidus muscle in patients with chronic low back pain. British Journal of Sports Medicine 2001 Jun;35(3):186-191.

D'Aprile P, Tarantino A, et al. The value of fat saturation sequences and contrast medium administration in MRI of degenerative disease of the posterior/perispinal elements of the lumbosacral spine. Eur Radiol 2007;17(2): 523-531.

De Coninck K, Passfield L, et al. An ultrasound evaluation of the relationship between changes in the lumbar perimuscular layer and Body Mass Index in people with non-specific lower back pain. J Bodyw Mov Ther. 2012;16(2):152-3.

Demir-Deviren S, Ozcan Eksi EE, Berven S. Patients with degenerative spondylolisthesis who chose to have surgery had significant instability and more fatty infiltration in erector spinae. PM and R. 2014;1:S366.

Demir-Deviren S, Ozcan Eksi EE Berven S. The effect of Modic 1 changes on paraspinal muscles in subjects with disc herniation. PM and R. 2014;1:S364.

D'Hooge R, Cagnie B, et al. Lumbar muscle dysfunction during remission of unilateral recurrent nonspecific low-back pain: Evaluation with muscle functional MRI. Clinical Journal of Pain 2013;29(3):187-194.

di Lorenzo L, Forte A, et al. Low back pain after unstable extracapsular hip fractures: randomized control trial on a specific training. Mediterranean Journal of Physical and Rehabilitation Medicine 2007 Sep;43(3):349-357.

Djordjevic O, Konstantinovic L, et al. Relationship Between Electromyographic Signal Amplitude and Thickness Change of the Trunk Muscles in Patients With and Without Low Back Pain. The Clinical journal of pain. 2015;31(10):893-902.

Dohzono S, Toyoda H, et al. Factors associated with improvement in sagittal spinal alignment after microendoscopic laminotomy in patients with lumbar spinal canal stenosis. J Neurosurg Spine. 2016;25(1):39-45.

Druschel C, Holzwarth F, et al. OPS-Imaging of human skeletal muscle microcirculation: Comparison of erector spinae muscle microvascular perfusion, fatty degeneration and postoperative clinical outcome in chronic back pain patients. Eur Spine J. 2010;19 (11):2034-5.

Druschel C, Zeitler S, et al. Changes of the ROM following mono-or bisegmental lumbar spinal fusions. Eur Spine J. 2010;19 (11):1978.

Ekin EE, Kurtul Yildiz H, Mutlu H. Age and sex-based distribution of lumbar multifidus muscle atrophy and coexistence of disc hernia: an MRI study of 2028 patients. Diagn Interv Radiol. 2016 [epub] [accepted for publication 29/09/15].

Fan SW, Hu ZJ, et al. Comparison of paraspinal muscle injury in one-level lumbar posterior inter-body fusion: modified minimally invasive and traditional open approaches. Orthopaedic surgery 2010;2(3): 194-200.

Fidler MW, Jowett RL. Muscle imbalance in the aetiology of scoliosis. J Bone Joint Surg Br 1976;58(2): 200-201.

Fidler MW, Jowett RL, et al. Myosin ATPase activity in multifidus muscle from cases of lumbar spinal derangement. J Bone Joint Surg Br 1975;57(2): 220-227.

Fischer MA, Nanz D, et al. Quantification of muscle fat in patients with low back pain: Comparison of multi-echo MR imaging with single-voxel MR spectroscopy. Radiology 2013;266(2): 555-563.

Fortin M. Paraspinal muscle morphology, composition and asymmetries: determinants and relation to low back pain and pathology. Ph.D. thesis 2013:199.

Fortin M, Gibbons LE, et al. Do variations in paraspinal muscle morphology and composition predict low back pain in men? Scand J Med Sci Sports. 2015;25(6):880-7.

Fortin M, Lazary A, et al. Association between paraspinal muscle morphology, clinical symptoms and functional status in patients with lumbar spinal stenosis. Eur Spine J. 2017;25(5):1452-9.

Franke J, Hesse T, et al. Morphological changes of the multifidus muscle in patients with symptomatic lumbar disc herniation: Clinical article. Journal of Neurosurgery: Spine 2009;11(6): 710-714.

Froholdt A, Holm I, et al. No difference in long-term trunk muscle strength, cross-sectional area, and density in patients with chronic low back pain 7 to 11 years after lumbar fusion versus cognitive intervention and exercises. The Spine Journal 2011 Aug;11(8):718-725.

Gejo R, Matsui H, et al. Serial changes in trunk muscle performance after posterior lumbar surgery. Spine (Phila Pa 1976) 1999;24(10): 1023-1028.

Gellhorn AC, Suri P, et al. Lumbar Muscle Cross-Sectional Areas Do Not Predict Clinical Outcomes in Adults With Spinal Stenosis: A Longitudinal Study. PM & R : the journal of injury, function, and rehabilitation. 2017;9(6):545-55.

Ghiasi MS, Arjmand N, et al. Cross-sectional area of human trunk paraspinal muscles before and after posterior lumbar surgery using magnetic resonance imaging. Eur Spine J. 2016;25(3):774-82.

Gille O, Jolivet E, et al. Erector spinae muscle changes on magnetic resonance imaging following lumbar surgery through a posterior approach. Spine (Phila Pa 1976) 2007;32(11): 1236-1241.

Gombatto SP, Norton BJ, et al. Factors contributing to lumbar region passive tissue characteristics in people with and people without low back pain. Clin Biomech (Bristol, Avon) 2013;28(3): 255-261.

Gordon TP, Sage MR, et al. Computed tomography of paraspinal musculature in ankylosing spondylitis . J Rheumatol 1984;11(6): 794-797.

Gornet MF, Schranck F, et al. Optimizing success with lumbar disc arthroplasty. Eur Spine J 2014;23(10): 2127-2135.

Hagg O, Fritzell P, et al. Predictors of outcome in fusion surgery for chronic low back pain. A report from the Swedish Lumbar Spine Study. Eur Spine J 2003 Feb;12(1):22-33.

Hansen BB, Bendix T, et al. Effect of lumbar disc degeneration and low-back pain on the lumbar lordosis in supine and standing: A cross-sectional MRI study. Spine (Phila Pa 1976). 2015;40(21):1690-6.

Hartwig T, Disch A, et al. A prospective analysis of quantitative changes of the lumbar paravertebral muscle volumes after MR-guided radiofrequency neurotomy of lumbar facet joints. Eur Spine J. 2012;21(11):2366.

Hicks G, Simonsick E, et al. Trunk muscle composition as a predictor of reduced functional capacity in the health, aging and body composition study: the moderating role of back pain. J Gerontol A Biol Sci Med Sci 2005;60(11): 1420-1424.

Hides J, Stanton W, et al. Effects of stabilization training on multifidus muscle cross-sectional area among young elite cricketers with low back pain. Journal of Orthopaedic & Sports Physical Therapy 2008;38(3): 101-108.

Hides J, Stanton W, et al. Effect of stabilisation training on trunk muscle size, motor control, low back pain and player availability among elite australian rules football players. Br J Sports Med 2011;45(4): 320-320.

Hides JA, Richardson CA, Jull GA. Multifidus muscle recovery is not automatic after resolution of acute, first-episode low back pain. Spine 1996 Dec 1;21(23):2763-2769.

Hirano S. Electron microscopic studies on back muscles in scoliosis. Nihon Seikeigeka Gakkai Zasshi 1972;46(1): 47-62.

Holt J, Macias B, et al. WISE 2005: Aerobic and resistive countermeasures prevent paraspinal muscle deconditioning during 60-day bed rest in women. Appl Physiol. 2016;120(10):1215-22.

Ho-Pham LT, Lai TQ, et al. Prevalence and pattern of radiographic intervertebral disc degeneration in Vietnamese: a population-based study. Calcif Tissue Int. 2015;96(6):510-7.

Hoshikawa Y, Iida T, et al. Effects of stabilization training on trunk muscularity and physical performances in youth soccer players. Journal of Strength and Conditioning Research 2013;27(11): 3142-3149.

Hosseinifar M, Akbari M, et al. The effects of stabilization and McKenzie exercises on transverse abdominis and multifidus muscle thickness, pain, and disability: a randomized controlled trial in nonspecific chronic low back pain. Journal of Physical Therapy Science 2013 Dec;25(12):1541-1545.

Huang Q, Li D, et al. Comparison of the Efficacy of Different Long-term Interventions on Chronic Low Back Pain Using the Cross-sectional Area of the Multifidus Muscle and the Thickness of the Transversus Abdominis Muscle as evaluation indicators. J Phys Ther Sci 2014;26(12): 1851-1854.

Huang QC, Li DS, et al. The Intervention Effects of Different Treatment for Chronic Low Back Pain as Assessed by the Cross-sectional Area of the Multifidus Muscle. J Phys Ther Sci 2013;25(7): 811-813.

Hung CW, Wu MF, et al. Comparison of multifidus muscle atrophy after posterior lumbar interbody fusion with conventional and cortical bone trajectory. Clin Neurol Neurosurg. 2016;145:41-5.

Hyun SJ, Kim YB, et al. Postoperative changes in paraspinal muscle volume: Comparison between paramedian interfascial and midline approaches for lumbar fusion. J Korean Med Sci 2007;22(4): 646-651.

Iwai K, Koyama K, et al. Asymmetrical and smaller size of trunk muscles in combat sports athletes with lumbar intervertebral disc degeneration. SpringerPlus. 2016;5(1):1474.

Jeon IC, Kwon OY, et al. Comparison of psoas major muscle thickness measured by sonography during active straight leg raising in subjects with and without uncontrolled lumbopelvic rotation. Man Ther. 2016;21:165-9.

Jinkins JR. Lumbosacral interspinous ligament rupture associated with acute intrinsic spinal muscle degeneration. Eur Radiol 2002;12(9): 2370-2376.

Joseph LH, Hussain RI, et al. Pattern of changes in local and global muscle thickness among individuals with sacroiliac joint dysfunction. Hong Kong Physiotherapy Journal. 2015;33(1):28-33.

Kader D, Radha S, et al. Evaluation of perifacet injections and paraspinal muscle rehabilitation in treatment of low back pain. A randomised controlled trial. Ortopedia, Traumatologia, Rehabilitacja 2012 May-Jun;14(3):251-259.

Kader DF, Wardlaw D, Smith FW. Correlation between the MRI changes in the lumbar multifidus muscles and leg pain. Clin Radiol 2000;55(2): 145-149.

Kalichman L, Carmeli E, Been E. The Association between Imaging Parameters of the Paraspinal Muscles, Spinal Degeneration, and Low Back Pain. 2017;2017:2562957.

Kanbara S, Yukawa Y, et al. Surgical outcomes of modified lumbar spinous process-splitting laminectomy for lumbar spinal stenosis. J Neurosurg Spine: 2015;22:353-7.

Kaser L, Mannion AF, et al. Active therapy for chronic low back pain. Part 2. effects on paraspinal muscle cross- sectional area, fiber type size, and distribution [with consumer summary]. Spine 2001 Apr 15;26(8):909-919.

Keller A, Brox JI, et al. Trunk muscle strength, cross-sectional area, and density in patients with chronic low back pain randomized to lumbar fusion or cognitive intervention and exercises [with consumer summary]. Spine 2004 Jan 1;29(1):3-8.

Keller A, Brox JI, Reikeras O. Predictors of change in trunk muscle strength for patients with chronic low back pain randomized to lumbar fusion or cognitive intervention and exercises. Pain Medicine 2008;9(6): 680-687.

Keller A, Johansen JG, et al. Predictors of isokinetic back muscle strength in patients with low back pain. Spine (Phila Pa 1976) 1999;24(3): 275-280.

Ketelhut NB, Kindred JH, et al. Core muscle characteristics during walking of patients with multiple sclerosis. J Rehabil Res Dev. 2015;52(6):713-24.

Khan AA, Iliescu DD, et al. Principal component and factor analysis to study variations in the aging lumbar spine. IEEE journal of biomedical and health informatics. 2015;19(2):745-51.

Khosla S, Tredwell SJ, et al. An ultrastructural study of multifidus muscle in progressive idiopathic scoliosis. Changes resulting from a sarcolemmal defect at the myotendinous junction. J Neurol Sci 1980;46(1): 13-31.

Kiesel KB, Underwood FB, et al. A comparison of select trunk muscle thickness change between subjects with low back pain classified in the treatment-based classification system and asymptomatic controls. J Orthop Sports Phys Ther 2007;37(10): 596-607.

Kim DY, Lee SH, et al. Comparison of multifidus muscle atrophy and trunk extension muscle strength - Percutaneous versus open pedicle screw fixation. Spine (Phila Pa 1976) 2005;30(1): 123-129.

Kim GY, Kin SH. Effects of Push-ups Plus Sling Exercise on Muscle Activation and Cross-sectional Area of the Multifidus Muscle in Patients with Low Back Pain. J Phys Ther Sci 2013;25(12): 1575-1578.

Kim JY, Ryu DS, et al. Paraspinal muscle, facet joint, and disc problems: risk factors for adjacent segment degeneration after lumbar fusion. The spine journal: official journal of the North American Spine Society. 2016;16(7):867-75.

Kim K, Isu T, et al. Comparison of the effect of 3 different approaches to the lumbar spinal canal on postoperative paraspinal muscle damage. Surgical Neurology 2008;69(2):109-113

Kim S, Kim H, Chung J. Effects of Spinal Stabilization Exercise on the Cross-sectional Areas of the Lumbar Multifidus and Psoas Major Muscles, Pain Intensity, and Lumbar Muscle Strength of Patients with Degenerative Disc Disease. J Phys Ther Sci 2014;26(4): 579-582.

Kong MH, Morishita Y, et al. Lumbar segmental mobility according to the grade of the disc, the facet joint, the muscle, and the ligament pathology by using kinetic magnetic resonance imaging. Spine (Phila Pa 1976) 2009;34(23): 2537-2544.

Kotilainen E, Alanen A, et al. Cross-sectional areas of lumbar muscles after surgical treatment of lumbar disc herniation. A study with magnetic resonance imaging after microdiscectomy or percutaneous nucleotomy. Acta Neurochirurgica 1995;133(1-2): 7-12.

Kraft CN, Pennekamp PH, et al. Magnetic resonance imaging findings of the lumbar spine in elite horseback riders: Correlations with Back pain, body mass index, trunk/leg-length coefficient, and riding discipline. American Journal of Sports Medicine 2009;37(11): 2205-2213.

Kulig K, Scheid AR, et al. Multifidus morphology in persons scheduled for single-level lumbar microdiscectomy: qualitative and quantitative assessment with anatomical correlates. American Journal of Physical Medicine & Rehabilitation 2009;88(5): 355-361.

Laasonen EM. Atrophy of sacrospinal muscle groups in patients with chronic, diffusely radiating lumbar back pain. Neuroradiology 1984;26(1): 9-13.

Le Cara EC, Marcus RL, et al. Morphology versus function: The relationship between lumbar multifidus intramuscular adipose tissue and muscle function among patients with low back pain. Arch Phys Med Rehabil 2014;95(10): 1846-1852.

Le Huec JC, Basso Y, et al. Influence of facet and posterior muscle degeneration on clinical results of lumbar total disc replacement: two-year follow-up. J Spinal Disord Tech 2005;18(3): 219-223.

Lee HJ, Lim WH, et al. The Relationship between Cross Sectional Area and Strength of Back Muscles in Patients with Chronic Low Back Pain. Ann Rehabil Med 2012;36(2): 173-181.

Lee JH, Lee SH. Does lumbar paraspinal muscles improve after corrective fusion surgery in degenerative flat black? BioMed research international. 2017;51(2):147-54.

Lee SH, Park SW, et al. The fatty degeneration of lumbar paraspinal muscles on computed tomography scan according to age and disc level. The spine journal: official journal of the North American Spine Society. 2017;17(1):81-7.

Lee W, Lee Y, Gong W. The Effect of Lumbar Strengthening Exercise on Pain and the Cross-sectional Area Change of Lumbar Muscles. J Phys Ther Sci 2011;23(2): 209-212.

Lehto M, Hurme M, et al. Connective tissue changes of the multifidus muscle in patients with lumbar disc herniation. An immunohistologic study of collagen Types I and III and fibronectin. Spine (Phila Pa 1976) 1989;14(3): 302-309.

Maffulli N. Histochemical and physiological studies in idiopathic scoliosis. Ital J Orthop Traumatol 1990;16(1):61-71.

Mannion AF, Dvorak J, et al. Increase in strength after active therapy in chronic low back pain (CLBP) patients: muscular adaptations and clinical relevance. Schmerz 2001;15(6): 468-473.

Mannion AF, Kaser L, et al. Influence of age and duration of symptoms on fibre type distribution and size of the back muscles in chronic low back pain patients. Eur Spine J 2000;9(4): 273-281.

Margraf NG, Rohr A, et al. MRI of lumbar trunk muscles in patients with Parkinson's disease and camptocormia. J Neurol. 2015;262(7):1655-64.

Masaki M, Aoyama T, et al. Association of low back pain with muscle stiffness and muscle mass of the lumbar back muscles, and sagittal spinal alignment in young and middle-aged medical workers. Clin Biomech (Bristol, Avon). 2017;49:128-33.

Mawston G, Boocock M. Lumbar posture biomechanics and its influence on the functional anatomy of the erector spinae and multifidus. Phys Ther Rev. 2015;20(3):178-86.

Mehta R, Cannella M, et al. Trunk Postural Muscle Timing Is Not Compromised In Low Back Pain Patients Clinically Diagnosed With Movement Coordination Impairments. Motor Control. 2017;21(2):133-57.

Millner J, Hides J, et al. The lumbo-pelvic muscles and ankylosing spondylitis: An observational pilot study. Intern Med J. 2012;42(S1):7.

Min SH, Kim MH, et al. The quantitative analysis of back muscle degeneration after posterior lumbar fusion: comparison of minimally invasive and conventional open surgery. Asian Spine J 2009;3(2): 89-95.

Moal B, Bronsard N, et al. Evaluation of muscle volume and fat infiltration of the Lumbar-Pelvic-Femoral complex in adult spinal deformity. Eur Spine J. 2013;22 (5):1217.

Mooney V, Gulick J, et al. Relationships between myoelectric activity, strength, and MRI of lumbar extensor muscles in back pain patients and normal subjects. Journal of Spinal Disorders 1997;10(4): 348-356.

Mori E, Okada S, et al. Spinous process-splitting open pedicle screw fusion provides favorable results in patients with low back discomfort and pain compared to conventional open pedicle screw fixation over 1 year after surgery. Eur Spine J. 2012;21(4): 745-753.

Motosuneya T, Asazuma T, et al. Postoperative change of the cross-sectional area of back musculature after 5 surgical procedures as assessed by magnetic resonance imaging. J Spinal Disord Tech 2006;19(5): 318-322.

Nabavi N, Mohseni Bandpei MA, et al. The Effect of 2 Different Exercise Programs on Pain Intensity and Muscle Dimensions in Patients With Chronic Low Back Pain: A Randomized Controlled Trial. J Manipulative Physiol Ther. 2017.

Niemelainen R, Briand MM, Battie MC. Substantial asymmetry in paraspinal muscle cross-sectional area in healthy adults questions its value as a marker of low back pain and pathology. Spine (Phila Pa 1976) 2011;36(25): 2152-2157.

Ntilikina Y, Bahlau D, et al. Open versus percutaneous instrumentation in thoracolumbar fractures: magnetic resonance imaging comparison of paravertebral muscles after implant removal. J Neurosurg Spine. 2017;27(2):235-41.

Ozcan E, Berven S, et al. The effect of paraspinal muscles on the improvement of patients with degenerative spondylolisthesis after surgery. PM and R. 2014;1:S364.

Prasarn ML, Rechtine GR, et al. Does lumbar paraspinal muscle fatty degeneration correlate with aerobic index and oswestry disability index? Surg Neurol Int. 2015; 6(Suppl 4): S240-S243.

Rantanen J, Hurme M, et al. The lumbar multifidus muscle five years after surgery for a lumbar intervertebral disc herniation. Spine (Phila Pa 1976) 1993;18(5): 568-574.

Regev GJ, Kim CW, et al. Regional myosin heavy chain distribution in selected paraspinal muscles. Spine (Phila Pa 1976) 2010;35(13): 1265-1270.

Saifuddin A, Sherazi Z, et al. Spinal osteoblastoma: relationship between paravertebral muscle abnormalities and scoliosis. Skeletal Radiol 1996;25(6): 531-535.

Saka K. Biomechanical analysis of scoliosis and back muscles using CT evaluation and the finite element method. Nihon Seikeigeka Gakkai Zasshi 1987;61(4): 299-310.

Salminen JJ, Erkintalo-Tertti MO, Paajanen HE. Magnetic resonance imaging findings of lumbar spine in the young: correlation with leisure time physical activity, spinal mobility, and trunk muscle

strength in 15-year-old pupils with or without low-back pain. Journal of Spinal Disorders 1993;6(5): 386-391.

Sanjana F, Chaudhry H, Findley T. Effect of MELT method on thoracolumbar connective tissue: The full study. J Bodyw Mov Ther. 2017;21(1):179-85.

Shahidi B, Hubbard JC, et al. Lumbar multifidus muscle degenerates in individuals with chronic degenerative lumbar spine pathology. J Orthop Res. 2017.

Shahidi B, Parra CL, et al. Contribution of Lumbar Spine Pathology and age to Paraspinal Muscle Size and fatty Infiltration. Spine (Phila Pa 1976). 2016 10.1097/brs.0000000000001848.

Sions J, Coyle P, et al. Multifidi Muscle Characteristics and Physical Function Among Older Adults With and Without Chronic Low Back Pain. Arch Phys Med Rehabil. 2017;98(1):51-7.

Sions J, Teyhen D, Hicks G. Criterion Validity of Ultrasound Imaging: Assessment of Multifidi Cross-Sectional Area in Older Adults With and Without Chronic Low Back Pain. Journal of geriatric physical therapy (2001). Epub Dec 23 2015.

Slager UT, Hsu JD. Morphometry and pathology of the paraspinous muscles in idiopathic scoliosis. Developmental Medicine and Child Neurology 1986:28(6): 749-756.

Smuck M, Crisostomo RA, et al. Morphologic changes in the lumbar spine after lumbar medial branch radiofrequency neurotomy: a quantitative radiological study. The spine journal: official journal of the North American Spine Society. 2015;15(6):1415-21.

Sokunbi O, Watt P, Moore A. A randomised controlled trial (RCT) on the effects of frequency of application of spinal stabilisation exercises on multifidus cross sectional area (MFCSA) in participants with chronic low back pain. Physiotherapy Singapore 2008 Jun;11(2):9-16.

Spencer GS, Eccles MJ. Spinal muscle in scoliosis. Part 2. The proportion and size of type 1 and type 2 skeletal muscle fibres measured using a computer-controlled microscope. J Neurol Sci 1976;30(1):143-154.

Storheim K, Berg L, et al. Fat in the lumbar multifidus muscles - predictive value and change following disc prosthesis surgery and multidisciplinary rehabilitation in patients with chronic low back pain and degenerative disc: 2-year follow-up of a randomized trial. BMC Musculoskelet Disord. 2017;18(1):145.

Storheim K, Holm I, et al. The effect of comprehensive group training on cross-sectional area, density, and strength of paraspinal muscles in patients sick-listed for subacute low back pain. J Spinal Disord Tech. 2003 Jun;16(3):271-279.

Strube P, Putzier M, et al. Postoperative posterior lumbar muscle changes and their relationship to segmental motion preservation or restriction: a randomized prospective study. J Neurosurg Spine. 2016;24(1):25-31.

Suh DW, Kim Y, et al. Reliability of histographic analysis for paraspinal muscle degeneration in patients with unilateral back pain using magnetic resonance imaging. J Back Musculoskelet Rehabil. 2017;30(3):403-12.

Tabaraee E, Ahn J, et al. Quantification of Multifidus Atrophy and Fatty Infiltration Following a Minimally Invasive Microdiscectomy. International journal of spine surgery. 2015;9:25.

Takayama K, Kita T, et al. A new predictive index for back muscle degeneration and sagittal alignment associated with aging. Eur Spine J. 2013;1:S688.

Tanida S, Fujibayashi S, et al. Influence of spinopelvic alignment and morphology on deviation in the course of the psoas major muscle. J Orthop Sci. 2017.

Teichtahl AJ, Urquhart DM, et al. Fat infiltration of paraspinal muscles is associated with low back pain, disability, and structural abnormalities in community-based adults. Spine J 2015 (epub) http://dx.doi.org/10.1016/j.spinee. 2015.03.039

Teichtahl AJ, Urquhart DM, et al. Lumbar disc degeneration is associated with modic change and high paraspinal fat content - a 3.0T magnetic resonance imaging study. BMC Musculoskelet Disord. 2016;17(1):439.

Teruo K, Takayama K, et al. Aging-associated changes in paraspinal muscles and psoas muscles. Eur Spine J. 2013;1:S683.

Tonomura H, Hatta Y, et al. Magnetic Resonance Imaging Evaluation of the Effects of Surgical Invasiveness on Paravertebral Muscles After Muscle-preserving Interlaminar Decompression (MILD). Clinical spine surgery. 2017;30(2):E76-e82.

Toyone T, Ozawa T, et al. No correlation found between patient outcome and abnormal lumbar mri findings indicating paraspinal muscle damage 5 years after decompressive unilateral procedure for lumbar canal stenosis. Spine (Phila Pa 1976). 2010 Conference publication.

Traistaru R, Popescu R, et al. Complex assessment of chronic back pain in patients with degenerative lumbar spondylolisthesis. Poster presentation. Osteoporos Int. 2011;22:S183-S4.

Traistaru R, Rogoveanu O, et al. Complex assessment of osteoporotic females with degenerative lumbar spondylolisthesis. Poster presentation. Osteoporos Int. 2014;25:S223.

Tsutsumimoto T, Shimogata M, et al. Mini-open versus conventional open posterior lumbar interbody fusion for the treatment of lumbar degenerative spondylolisthesis: Comparison of paraspinal muscle damage and slip reduction. Spine (Phila Pa 1976) 2009;34(18): 1923-1928.

Verla T, Adogwa O, et al. Effects of Psoas Muscle Thickness on Outcomes of Lumbar Fusion Surgery. World Neurosurg. 2016;87:283-9.

Waschke A, Hartmann C, et al. Denervation and atrophy of paraspinal muscles after open lumbar interbody fusion is associated with clinical outcome-electromyographic and CT-volumetric investigation of 30 patients. Acta Neurochirurgica 2014;156(2): 235-244.

Weber BR, Grob D, et al. Posterior surgical approach to the lumbar spine and its effect on the multifidus muscle. Spine (Phila Pa 1976) 1997;22(15): 1765-1772.

Whalen RG, Ecob MS. Two-dimensional electrophoretic analysis of muscle contractile proteins in patients with idiopathic scoliosis. Clin Chem 1982;28(4 Pt 2): 1036-1040.

Willemink MJ, van Es HW, et al. The effects of dynamic isolated lumbar extensor training on lumbar multifidus functional cross-sectional area and functional status of patients with chronic nonspecific low back pain. Spine (Phila Pa 1976) 2012;37(26): E1651-1658.

Wong AYL, Parent EC, et al. Do participants with low back pain who respond to spinal manipulative therapy differ biomechanically from nonresponders, untreated controls or asymptomatic controls? Spine (Phila Pa 1976). 2015;40(17):1329-37.

Wu W, Hu Z, et al. Influencing of chronic low back pain on multifidus muscle atrophy. China journal of orthopaedics and traumatology 2014;27(3): 207-212.

Xu WB, Chen S, et al. Facet orientation and tropism: Associations with asymmetric lumbar paraspinal and psoas muscle parameters in patients with chronic low back pain. J Back Musculoskelet Rehabil. 2016;29(3):581-6.

Yarom R, Robin GC. Muscle pathology in idiopathic scoliosis. Isr J Med Sci 1979;15(11):917-924.

Yoo JS, Min SH, et al. Paraspinal muscle changes of unilateral multilevel minimally invasive transforaminal interbody fusion. J Orthop Surg Res 2014;9:130.

Zapata KA, Wang-Price SS, et al. Ultrasonographic measurements of paraspinal muscle thickness in adolescent idiopathic scoliosis: a comparison and reliability study. Pediatr Phys Ther. 2015;27(2):119-25.

Zetterberg C, Aniansson A, Grimby G. Morphology of the paravertebral muscles in adolescent idiopathic scoliosis. Spine (Phila Pa 1976) 1983;8(5): 457-462.

Zhu W, Zang L, et al. Correlation between multifidus muscle and proximal junctional kyphosis after long-segment instrumentation for lumbar degenerative disease. Int J Clin Exp Med. 2017;10(6):9463-9.

Zhu XZ, Parnianpour M, et al. Histochemistry and morphology of erector spinae muscle in lumbar disc herniation. Spine (Phila Pa 1976) 1989;14(4): 391-397.

Zlomislic V, Allen RT, et al. Lumbar muscle structure and function in chronic versus recurrent low back pain: a cross-sectional study. J Orthop Res. 2017;17(9):1285-96.

Zoabli G, Mathieu PA, Aubin CE. Back muscles biometry in adolescent idiopathic scoliosis. Spine Journal 2007;7(3): 338-344.

Zotti MG, Boas FV, et al. Does pre-operative magnetic resonance imaging of the lumbar multifidus muscle predict clinical outcomes following lumbar spinal decompression for symptomatic spinal stenosis? Eur Spine J. 2017 10.1007/s00586-017-4986-x.
