# Supplementary material for: Relationships between paraspinal muscle morphology and neurocompressive conditions of the lumbar spine: a systematic review with meta-analysis
Source: BMC Musculoskelet Disord. 2018 Sep 27;19:351. doi: 10.1186/s12891-018-2266-5 (PMC6161433; doi:10.1186/s12891-018-2266-5)
Supplement: Supplementary file 5 — Extracted data and outcomes broken down by pathology and assessment methodology† (PDF 174 kb) [file 12891_2018_2266_MOESM5_ESM.pdf]

Appendix 5 Extracted data and outcomes broken down by pathology and assessment methodology†

**Assessing muscle changes associated with disc herniation via imaging**				
**Study**	**Study population**	**Comparison / control**	**Assessment method / variables**	**Study results**
Altinkaya [35]<br>[Cross-sectional study]	122 pre-surgical university hospital patients with unilateral, single-level LDH (L3/4 – L5/S1)<br><br>M/F = 64/58; mean age: 46 years ± 12.7 (16 - 80 years)<br><br>Stratified by symptom duration:<br>A: ≤ 30 days: 48<br>B: ≤ 90 days: 26<br>C: > 90 days: 48	Patients served as own controls [affected vs. unaffected side analysis]	1.5T MRI, T2 axial images assessing TCSA (excluding centrally located fat but including intramuscular fat) and MLD of the LMM bilaterally at level of herniation (if foraminal) or at the level directly below herniation (if recessal)	**Duration of symptoms:**<br>Median CSA smaller on diseased side for each group, but not significantly (p>0.05 between groups) and smaller as chronicity increases (no p value reported); median MLD significantly larger on diseased side for each group (p<0.05 between groups), and larger as chronicity increases (p=0.021)<br><br>Median MLD (mm) (Min-Max): [D=Diseased / C=control]<br>AD: 5.1 (2.3–12.8) / AC: 4.8 (2.5–12.4)<br>BD: 6.7 (3.0–11.2) / BC: 5.4 (2.8–10.7)<br>CD: 7.6 (3.3–13.7) / CC: 5.0 (2.1–12.0)<br><br>**Severity of compression:**<br>CSA smaller & MLD larger on diseased side (p>0.05 between groups & sides).<br><br>Median MLD (mm) (Min-Max): [D=Diseased; C=control; 1,2,3 = grades of NR compression]<br>1D: 4.9 (3.4–8.1) / 1C: 4.9 (2.9–8.3)<br>2D: 6.5 (2.3–13.7) / 2C: 4.9 (2.1–12.4)<br>3D: 6.7 (3.0–13.4) / 3C: 4.9 (2.5–11.4)
Battie [15]<br>[Cross-sectional study]	43 patients with unilateral radiculopathy and MRI confirmed ipsilateral, single level LDH at L4 (11) or L5 (32)<br><br>M/F = 32/11; mean age: 41 years (22 - 63)<br><br>Leg symptoms on L/R side = 27/16;<br><6 weeks from symptom onset to imaging	Patients served as own controls [affected vs. unaffected side analysis]	1.5T MRI, T2 axial images assessing TCSA, FCSA, FCSA:TCSA ratio & total muscle signal intensity of LMM (primary) and ESM / PMM (secondary) bilaterally at L3/4, L4/5, L5/S1, and mid S1 levels [For the "level below LDH" results included, all measurements were acquired at the level directly below the LDH]	**LMM**: Significant TCSA asymmetry only apparent at the level of LDH. Fat content higher on affected side at level below LDH, but FCSA asymmetry not significantly different. FCSA:TCSA ratio significantly smaller on affected side at and below level of LDH. Multifidus signal intensity significantly higher on affected side at level below LDH only.<br><br>**LMM TCSA:** (both levels combined)<br>At level of LDH: affected side: 9.9cm^2 (2.2cm^2); unaffected side: 9.5cm^2 (2.0cm^2) *p=0.033*<br><br>**LMM FCSA:** (both levels combined)<br>At level below LDH: affected side: 7.6cm^2 (2.3cm^2); unaffected side: 7.9cm^2 (2.0cm^2) *p=0.133*<br><br>**LMM FCSA:TCSA ratio:** (combined)<br>At level of LDH: affected side: 0.78 (0.09); unaffected side: 0.80 (0.07) *p=0.031*<br>At level below LDH: affected side: 0.69 (0.12); unaffected side: 0.72 (0.09) *p=0.007*<br><br>**LMM signal** (combined)<br>At level below LDH: affected side: 153 (60); unaffected side: 142 (49) *p=0.014*<br><br>**ESM/ PMM:** Significantly higher ESM signal noted at level of LDH only. No significant difference of TCSA, FCSA, or F/T ratio for ESM (p range = 0.078-0.934) or PMM CSA (p range = 0.399-0.639) shown.<br><br>**ESM signal:** (combined)<br>At level of LDH: affected: 145 (63); unaffected: 137 (56) *p=0.017*

Study	Study population	Comparison / control	Assessment method / variables	Study results
Bhadresha [40]<br><br>[Cross-sectional study]	107 pre-operative spine surgeon patients with history of radicular pain due to LDH, one or more levels, between L3-S1<br><br>M/F = 59/48; mean age: 39.1 years; VAS (back): 6.6 (6.2-7.1) / (leg): 6.7 (6.1-7.2); ODI (%): 45.8 (4.1-49.5)	58 LBP patients with DDD at one or more levels, but no LDH<br><br>M/F = 19/39; mean age: 39.1 years; VAS (back): 7.4 (6.8-7.9) / (leg): 5.3 (4.7-6.0); ODI (%): 50.7 (45.7-55.7)	MRI, T2W axial images assessing the fat:muscle ratio of LMM, ESM & PMM (each combined bilaterally) from L3/4 - L5/S1 [NB: did not take side of radiculopathy into account]	**Muscle:fat ratio:** The muscle to fat ratio increased moving from L3 to L5 in the PMM, but decreased for LMM and ESM in both groups; however, no statistically significant differences in fat infiltration for any of the 3 muscle groups was noted at any level.<br><br>PMM: Mean range (DDD): 0.926-0.960; LDH: 0.938-0.953*; p range: 0.180 - 0.960*<br>ESM: Mean range (DDD): 0.650-0.812; LDH: 0.616-0.828; *p range: 0.082 – 0.445*<br>LMM: Mean range (DDD): 0.803-0.844; LDH: 0.805-0.833; *p range*: *0.532 – 0.879*<br><br>[Outcomes not matched to level of disc pathology – only to type of disc pathology]
Boyaci [36]<br><br>[Cross-sectional study]	32 physical medicine and rehabilitation clinic patients with LDH causing NR compression at L4/5<br><br>M/F=12/20; mean age: 48.4 ± 13.0 years<br><br>LBP > 1 year + 1 episode w/in last 6 months	28 patients from same source, without LDH [including patients with disc and facet joint degeneration without NR compression]<br><br>M/F=12/16; mean age: 43.8 ± 11.9 years.<br><br>LBP > 1 year + 1 episode w/in last 6 months	1.5T MRI, T2 axial images assessing TCSA & semi-quantitative muscle grading of the LMM, PVM (combined LMM/ ESM), QLM, & PMM bilaterally at L4/5	**TCSA**: significant difference in cross-sectional area of the right QLM, but not for any other muscle comparisons<br>LDH group: 417.7 ± 203.2mm^2<br>Control: 537.8±177.4mm^2 *p=0.01*<br><br>**Fat infiltration:** LDH group showed significantly different distribution of fat infiltration for all muscles groups except PMM (p range = 0.20-0.30), with higher grades more prevalent in LDH group.<br><br>[LDH:NonLDH]<br>R & L LMM: G1=13:20; G2=12:6; G3=7:2 (*p=0.04)*<br>R & L PVM: G1=11:19; G2=13:7; G3=8:2(*p=0.02)*<br>R & L QLM: G0=9:17; G1=17:9; G2=6:2 (*p=0.03)*<br><br>**Site of NR compression:** No correlation between bilateral vs. unilateral nerve root compression and cross-sectional area or fatty degeneration [correlation values range from -0.234 - 0.314; *all p values > 0.05]*.
Dangaria [37]<br><br>[Cross-sectional study]	25 hospital/ orthopaedic patients with intermittent or continuous unilateral sciatica (w/ or w/o LBP) and single level non-central LDH (10 at L4/5, 15 at L5/S1)<br><br>No patient demographics provided, but authors noted full compatibility of age, gender, weight, and height between groups<br><br>Median duration of symptoms 29.2 months (6 weeks - 6 years)	15 healthy hospital staff with no history of LBP or sciatica and normal lumbar MRIs<br><br>[Time period for recruitment between groups not indicated]<br>______________<br>Patients also served as own controls [affected vs. unaffected side analysis]	1.0T MRI, axial images assessing the TCSA of PMM bilaterally at L3/4, L4/5 and L5/S1 discs levels<br><br>*[Authors' contact details out of date; could not seek clarification on accuracy of mm^2 vs cm^2 TCSA values*]	**TCSA:** Measures were consistently smaller on the side of LDH at or below level of LDH for patients, but consistently symmetric in controls.<br><br>**LDH**: [median (range)(mm^2)][comparison is to LDH side, not level; spinal level not matched to LDH level]<br>R-sided herniation (12)<br>L4/5: R = 18.3 (10.3 - 24.4); L = 18.9 (11.1 - 25.9) *p<0.05*<br>L5/S1: R = 14.5 (7.4 - 26.9); L = 16.0 (8.5 - 25.4) *p<0.05*<br>L-sided herniation (13)<br>L4/5: L = 15.5 (8.1 - 27.6); R = 19.4 (8.3 - 29.3) *p<0.05*<br>L5/S1: L = 12.4 (5.3 - 18.4); R = 13.6 (6.0 - 25.9) *p<0.05*<br>[*query values: <25mm^2 for most CSAs seems quite small; may have been cm^2 measures*]<br><br>The percentage of reduction in TCSA on the side of LDH was only significant at the lower two levels.<br>L4/5: 8.6% (1.8 - 27.5) *p<0.05*<br>L5/S1: 8.4% (1.8 - 29.2) *p<0.05*<br><br>For the LDH group, there was positive correlation between the median duration of <u>continuous</u> sciatica and percentage reduction in TCSA on the affected side, but no correlation with intermittent sciatica.<br>L4/5: Spearman's rho = 0.8 *p=0.05*<br>L5/S1: Spearman's rho = 0.8 *p=0.03*

Study	Study population	Comparison / control	Assessment method / variables	Study results
Farshad [18]<br><br>[Cross-sectional study]	79 hospital spine clinic patients with unilateral, single level radiculopathy and MRI documented NR compression – assessed at all lumbar disc levels<br><br>M/F = 53/26; mean age: 48 ± 13 years (range 22 - 80)<br><br>Stratified by symptom duration:<br>1-30 days;<br>31-89 days;<br>>90 days	Patients served as own controls [affected vs. unaffected side analysis]	MRI, T2 axial images assessing LMM CSA ratios (functional muscle, including intramuscular fat) and MLD between the affected and contralateral side bilaterally, at and directly below the level of NR compression	**Severity**: There was no significant difference between the CSA or MLD ratios and severity of NR compression (p range = 0.577 – 0.664)<br><br>**Duration:** There was no significant relationship between the CSA or MLD ratios and symptom duration (p range = 0.292 – 0.894)<br><br>**Motor deficit:** there was no significant difference in CSA or MLD ratios noted by either examiner between those with and without motor deficit (p range = 0.568 – 0.584; 0.649 – 0.655)<br><br>**Side/level:** the overall CSA ratio was slightly smaller, and the MLD measurement significantly larger, on the pathologic vs. contralateral sides:<br>CSA ratio:<br>Examiner 1 = 0.997±0.152<br>Examiner 2 = 0.994±0.152<br><br>MLD: [compression side / contralateral side]<br>Examiner 1: 7.2±3.6 mm/6.6±3.1mm; *p=0.04*<br>Examiner 2: 7.0±3.6 mm/6.5±3.4mm; *p=0.09*
Fortin [41]<br><br>[Cross-sectional study]	33 patients with history of unilateral L4/5 LDH with radiculopathy drawn from lumbar pathology research consortium<br><br>M/F = 14/19; mean age: 48.8 ± 12.6 years; BMI: 27.5 ± 4.6<br><br>Mean symptom duration: 6.8 ± 16.6 months<br><br>Mean leg pain score (1-10): 7.5 ± 2.0; mean back pain score (1-10): 5.8 ± 2.8	Patients served as own controls [affected vs. unaffected side analysis]	MRI, T2W axial images assessing the TCSA, FCSA, FCSA:TCSA ratio, and signal intensity of the ESM and LMM bilaterally at L3/4, L4/5, L5/S1, and mid S1 levels	**LMM**: Although the trends were for larger TCSAs and smaller FCSAs & ratios on the affected side, no statistically significant side to side differences were found. LMM signal intensity was lower on the affected side at & above the level of LDH, but higher on the affected side below the level of LDH (but only significantly higher at L5/S1). No significant relationships between LMM asymmetry and symptom duration were noted.<br><br>**TCSA:** (*p range = 0.13 – 0.25*) / **FCSA:** (*p range = 0.40 – 0.69*)<br>**FCSA:TCSA ratio:** (*p range = 0.12 – 0.28*)<br>**Signal:**<br>At level below LDH (L5/S1): affected side: 2625.3 ± 2109.2; unaffected side: 2476.3 ± 1932.5 *p=0.04*<br><br>**ESM:** Except from a larger TCSA on the affected side at L3/4, the trend was towards smaller TCSAs & FCSAs on the affected side at all levels. Although no statistically significant side to side difference in the TCSA was found, the FCSA at L5/S1 and the ratios at L4/5 and L5/S1 were significantly smaller on the side of LDH. There was a trend towards higher signal intensity on the side of LDH at all levels, which was significant at S1. No significant relationships between ESM asymmetry and symptom duration were noted.<br><br>**TCSA:** (*p range = 0.05 – 0.94*)<br>**FCSA:** At level below LDH (L5/S1): affected side: 3.3 ± 2.5 cm^2; unaffected side: 4.0 ± 2.3 cm^2 *p=0.04*<br>**FCSA:TCSA ratio:**<br>At level of LDH: affected side: 0.47 ± 0.17; unaffected side: 0.52 ± 0.12 *p=0.04*<br>At level below LDH (L5/S1): affected side: 0.30 ± 0.15; unaffected side: 0.36 ± 0.15 *p=0.007*<br>**Signal:**<br>At level below LDH (S1): affected side: 3688.4 ± 2137.2; unaffected side: 3323.7 ± 1795.4 *p=0.02*

Study	Study population	Comparison / control	Assessment method / variables	Study results
Frost [42]<br>[Cross-sectional study]	17 chiropractic/ physiotherapy clinic LBP patients with lower lumbar LDH and unilateral radiculopathy<br>M/F = 6/11; mean age: 44.2 ± 14.9 years<br>Mean symptom duration: 126 ±143 months<br>VAS (back): 2.6 ± 2.3 / (leg): 1.8 ± 1.2; ODI %: 19.9 ± 12.8	17 healthy controls with no history of LBP or neurological deficit<br>All non-pain parameters matched to the primary group<br>——————<br>Patients also served as own controls [affected vs. unaffected side analysis]	High resolution axial DUS images assessing the echo intensity in the relaxed state of the ESM and LMM combined, at L2/3 bilaterally<br>[NB: measures taken at L2/3 while LDHs were all at or below L3/4]	**Fat infiltration:** The mean echo intensity did not demonstrate any significant between-group differences, or any significant correlations with duration or severity of symptoms<br>**Echo intensity:** [mean ± SE]<br>Control group: ~31.5 ± 4;<br>LDH - unaffected side: ~30 ± 4;<br>LDH - affected side: ~31.4 ± 4 $p>0.05$<br>[NB: measures estimated from graph]
Hyun [38]<br>[Cross-sectional study]	14 medical university patients with EMG-confirmed unilateral radiculopathy and MRI-confirmed LDH L3/4 – L5/S1<br>M/F = 9/5; mean age: 48.9 (± 15.4) years<br>Mean duration of symptoms: 16.5 (±33.5) months	25 comparison group patients similar to main group, but with unilateral radiculopathy ruled out by EMG<br>M/F = 14/11; mean age: 40.3 (± 14.5) years<br>Mean duration of symptoms: 7.6 (± 26.4) months<br>——————<br>20 control volunteers with no lumbar radiculo-pathy features or disc herniation<br>M/F = 13/7; mean age: 31.5 (± 9.3) years<br>——————<br>Both patient groups also served as own controls [affected vs. unaffected side analysis]	1.5T MRI, T1 axial images assessing TCSA, FCSA, muscle:total volume ratio, and functional muscle ratio between involved and uninvolved sides of the LMM bilaterally<br>[NB: measures not matched to specific level of LDH, but only to presence or absence of LDH affecting at least one level]	**Radiculopathy group:** There was no significant difference in TCSA or FCSA at any level between involved and uninvolved sides (p>0.05 all levels). However, the side-to-side FCSA ratios were significantly smaller at most levels and the percentage of patients with abnormal side-to-side FCSA ratios was significantly greater in this group.<br>**Involved:uninvolved side FCSA ratios:** [values estimated from graph]<br>L4–L5: 0.88 ± 0.15 (rad)* / 1.0 ± 0.10 (LDH) / 1.03 ± 0.05 (control) **p<0.01 Rad vs LDH/controls*<br>L5–S1: 0.83 ± 0.09 (rad)* / 0.98 ± 0.09 (LDH) / 1.03 ± 0.07 (control) **p<0.01 Rad vs LDH/controls*<br>All levels totalled: 0.9 ± 0.12 (rad)* / 1.0 ± 0.10 (LDH) / 1.03 ± 0.07 (control) **p<0.05 Rad vs LDH/ controls*<br>**Involved:uninvolved side ratio abnormal (% of subjects/group):**<br>Rad: 79%* / LDH: 24% / Control: 10% [**p<0.01 Rad vs LDH/controls*]<br>**LDH (no radiculopathy) group:** There was no significant difference in TCSA, FCSA, or F/T ratios at any level between involved and uninvolved sides in this group (p>0.05).<br>**Control group:** There was no significant difference in TCSA, FCSA, or F/T ratios between sides at any level within the control group (p>0.05 all levels). However, when comparing the control to the affected sides of the patient groups, the CSA was consistently and often significantly smaller in the two patient groups at L4/5 and L5/S1, with the radiculopathy group CSA being smallest across all categories.<br>**Control vs involved side of patient groups**:<br>[NB: for controls, TCSA & FCSA values for each side combined due to no significant difference noted]<br>**TCSA** (m ± sd; mm^2):<br>L5–S1: 586.78 ± 209.65* (rad); 696.58 ± 218.04* (LDH); 761.25 ± 196.34 (control)<br>**FCSA** (m ± sd; mm^2):<br>L4–L5: 440.17 ± 181.98* (rad); 507.82 ± 174.58* (LDH); 574.06 ± 132.61 (control)<br>L5–S1: 348.02 ± 240.44* (rad); 504.17 ± 226.52* (LDH); 569.37 ± 170.22 (control)<br>**FCSA:TCSA ratio** (m ± sd):<br>L3–L4: 0.79 ± 0.14* (rad); 0.81 ± 0.10* (LDH); 0.88 ± 0.09 (control)<br>L4–L5: 0.68 ± 0.23* (rad); 0.72 ± 0.13* (LDH); 0.81 ± 0.10 (control)<br>L5–S1: 0.55 ± 0.24 (rad); 0.70 ± 0.18 (LDH); 0.74 ± 0.11 (control) ** p<0.05 vs control*

Study	Study population	Comparison / control	Assessment method / variables	Study results
Kim [16]<br><br>[Cross-sectional study]	39 pre-surgical hospital patients with acute, unilateral leg pain due to L4/5 level LDH<br><br>M/F = 27/12; mean age: 42.2 ± 7.9 years (25 – 58 years); BMI 24.8 ± 2.5<br><br>Mean symptom duration: 0.6 ± 0.4 month (0.1 - 1 month)	37 patients with same criteria as primary group, but with chronic leg pain<br><br>M/F = 22/15; mean age: 44.6 ± 9.1 years (22-58 years); BMI 24.2 ± 3.2<br><br>Mean symptom duration: 5.4 ± 2.7 months (3-12 months)<br>_______________<br>Patients served as own controls [affected vs. unaffected side analysis]	1.5T MRI, T2 axial images assessing TCSA and the ratio of TCSA between diseased and normal sides for the LMM (excluding paralaminar fat) and PMM at the L4/5 disc level	**TCSA:** For the chronic group, the mean CSA of the LMM was significantly smaller on the side of LDH, but not for the acute group. For the PMM, neither group showed a significant difference in CSA (p range = 0.829 – 0.967).<br><br>LMM**:** (m ± sd; mm^2)<br>Acute:<br>Normal side: 664.7 ± 156.6<br>Diseased side: 680.1 ± 171.9 *p = 0.085*<br><br>Chronic:<br>Normal side: 675.3 ± 133.9<br>Diseased side: 632.9 ± 123.0 *p <0.01*<br><br>**Associations between CSA ratios and age, gender, BMI:** Weak and non-significant associations were found between CSA ratios and age, gender, BMI in both the acute and chronic groups.
Kong [39]<br><br>[Cross-sectional study]	40 pre-operative hospital patients with L4/5 LDH<br><br>M/F = 20/20; mean age: 41.9 ± 7.9 years; BMI: 23.4 ± 2.7	42 healthy/ normal subjects<br><br>M/F = 28/14; mean age: 42.2 ± 10.9 years; BMI: 22.8 ± 2.0<br><br>[No indication of population source of healthy subjects; time period of recruitment for this group not specified.]	MRI, T2W axial images assessing the TCSA and total fat CSA [*unclear if unilateral or bilateral*] of ESM and LMM (combined) from L2/3 - L5/S1<br><br>*[Attempted to contact main author to clarify which sides were measured – no reply received]*	**Fat infiltration:** The difference in infiltration rates in both groups increased significantly between spinal levels when moving caudally, being greatest in the LDH group. Mean fat infiltration per level was significantly greater at all levels in the LDH group.<br><br>Between levels**:** (difference ± sd, mm^2)<br>Normal group:<br>L2/3 - L3/4: -0.26 ± 1.98<br>L3/4 - L4/5: -1.37 ± 2.82*<br>L4/5 - L5/S1: -2.43 ± 3.03**<br><br>LDH group:<br>L2/3 - L3/4: -2.21 ± 3.24**<br>L3/4 - L4/5: -3.55 ± 3.35**<br>L4/5 - L5/S1: -4.62 ± 6.37**<br><br>Between groups**:** (m ± sd, mm^2)<br>L2/3: Normal: 3.71±2.81 / LDH: 5.52±3.68*<br>L3/4: Normal: 3.97±2.92 / LDH: 7.73±5.12**<br>L4/5: Normal: 5.35±3.97 / LDH: 11.28±6.13**<br>L5/S1: Normal: 7.79±3.52 / LDH: 15.9±7.19**<br><br>** p<0.05; ** p<0.001*

Study	Study population	Comparison / control	Assessment method / variables	Study results
Sun [43]<br><br>[Cross-sectional study]	60 hospital patients (Jan - April 2015) with isolated L4/5 LDH resulting in unilateral radiculopathy<br><br>M/F = 31/29; mean age: 48.97 ± 8.39 years [range: 32-65]<br><br>Mean symptom duration: 9.15 ± 5.51 months; VAS: 6.60 ± 1.33	60 hospital patients (Jan - April 2015) with non-specific LBP and no LDH<br><br>M/F = 29/31; mean age: 46.08 ± 7.27 years [range: 30-64]<br><br>[Symptom duration and VAS not provided]	MRI, T2W axial images assessing muscle atrophy via semi-quantitative grading of the LMM from L3/4 - L5/S1<br><br>*[Note: authors stated use of CSA to measure atrophy, but applied a fat infiltration grading system instead]*	Significantly greater and more severe muscle atrophy was noted in the LDH group vs control at all three spinal levels assessed.<br><br><u>Between groups</u>**:** (rank sum: LDH vs control)<br>L3/4: -5.126**<br>L4/5: -5.818**<br>L5/S1: -6.217**<br><br>** *$p<0.01$*

Assessing muscle changes associated with disc herniation via biopsy

Study	Study population	Comparison / control	Assessment method / variables	Study results
Bajek [44]<br><br>[Cross-sectional study]	76 hospital patients with clinical signs of NR compression, undergoing surgery for unilateral, single level LDH<br><br>M/F = 46/30; mean age: 47.2 years (27-67 years)<br><br>Symptom duration range: 6 months - 2.5 years (intermittent)<br><br>[No specific exclusion criteria provided]	41 deceased (sudden) persons, otherwise healthy with no neuromuscular disease<br><br>M/F = 35/6; mean age: 34.8 years (17-50 years)<br><br>Within 48 hrs of time of death<br><br>[No indication of time period for recruitment between groups]	Biopsy: histochemical analysis of Type I and II (A,B,C) fibers to assess % distribution and smaller fiber diameter of LMM at L3/4, L4/5, or L5/S1 (at level and on side of herniation); controls all assessed at L4/5	**Fiber type %:** There was a significantly higher % of Type I fibers and lower % of Type IIA and IIB fiber in men, but not in women, between the control and LDH groups.<br><br>Type I (men): [m ± sd]<br>Control: 60.76 ± 6.89; LDH: 67.4 ± 10.87 *$p<0.05$*<br>Type IIA (men):<br>Control: 21.72 ± 7.14; LDH: 18.16 ± 6.55 *$p<0.05$*<br>Type IIB (men):<br>Control: 17.48 ± 6.17; LDH: 14.23 ± 6.26 *$p<0.05$*<br><br>**Fiber type diameter (μm):** Type I fiber diameter was significantly larger in the LDH group for men and women, while both type II fibers were significantly larger in men.<br><br>Type I (men): [m ± sd]<br>Control: 62.16 ± 6.06; LDH: 69.94 ± 10.27 *$p<0.05$*<br>Type IIA (men):<br>Control: 49.67 ± 9.67; LDH: 56.02 ± 10.27 *$p<0.05$*<br>Type IIB (men):<br>Control: 42.85 ± 8.72; LDH: 48.27 ± 10.74 *$p<0.05$*<br><br>Type I (women):<br>Control: 53.92 ± 7.88; LDH: 69.61 ± 12.95 *$p<0.01$*<br><br>All other assessments: *$p>0.05$*

Study	Study population	Comparison / control	Assessment method / variables	Study results
Ford [45]<br>[Cross-sectional study]	18 hospital patients undergoing surgery for LDH, with positive clinical signs of unilateral NR involvement<br>[level of LDH not stated]<br>Age range: 28-73 years<br>Symptoms duration: 3 weeks - 1 year for all but one patient	Patients served as own controls [affected vs. unaffected side analysis]	Biopsy: Histo-chemical analysis assessing lesser fiber diameter, mean area of muscle cell, and muscle strength factor (% fiber type x mean fiber area) of the ESM/ sacro-spinalis (superficial) & LMM (deep) at L5 bilaterally	**Mean proportion of muscle fibers:**<br>No significant side-to-side differences were noted for any of the parameters analysed (*p>0.05)*.<br>**Mean size of muscle fibers (µm):**<br>No significant side-to-side differences were noted for any of the parameters analysed (*p>0.05)*.<br>**Mean strength factor:**<br>No significant side-to-side differences were noted for any of the parameters analysed (*p>0.05)*.<br>**Staining patterns:**<br>In muscle stained with NADH, no particular pattern was apparent in relation to either the site of sample, or the site and duration of protrusion (descriptive data only)
Mattila [46]<br>[Cross-sectional study]	41 hospital patients with LDH at L4 or L5, with surgically confirmed NR compression at L4/5 (41%), L5/S1 (24%), or both (34%)<br>M/F = 22/19; mean age: 42.2 ± 8.1 years (26-55 years)<br>Pre-operative sick leave days: 59.8 ± 56.5 (4-275 days)	12 autopsies (within 48 hrs of death); no known history of low back or general muscle disease (all but 2 being ambulatory at time of death)<br>M/F = 9/3; mean age: 40.5 years	Biopsy (2 per person): Histochemical analysis assessing atrophy and hypertrophy factors and ratios of mean size for type I and type II fibers at the transversospinal corner of the LMM at L4/5 and L5/S1 (both levels pooled)	For type I fibers, significant differences in diameter (males), hypertrophy factor (males), and core-targetoid (both) and moth-eaten (female) fiber frequencies were present between groups.<br>**Type I fiber type diameter (µm)** [m (sd)]:<br>Male: Patient: 59.7(13.6); Control: 51.2 (6.8) *p<0.01*<br>**Type I fiber hypertrophy factor** [m HF (sd)]:<br>Male: Patient: 232.6(313.8); Control: 68.5(88.2) *p<0.01*<br>**Core-targetoid change** [m % (sd)]:<br>Male: Patient: 4.7(8.3); Control: 0.3(0.5) *p<0.01*<br>Female: Patient: 4.9(6.2); Control: 0.0 (0.0) *p<0.001*<br>**Moth-eaten change** [m % (sd)]:<br>Female: Patient: 4.4(5.5); Control: 0.0(0.0) *p<0.001*<br>No significant differences were found for type I fiber diameter or atrophy factor, or for any type II fiber comparisons (*p>0.05)*<br>No significant correlations were noted for any factors against duration of sick leave.
Yoshihara [33]<br>[Cross-sectional study]	17 hospital patients with L4/5 LDH and unilateral lower limb sensory or motor impairment<br>M/F = 14/3; mean age: 35 years (21-54 years)<br>Mean interval from LDH to biopsy: 3 months (1-9 months)	Patients served as own controls [affected vs. unaffected side analysis]	Biopsy: Histochemical analysis assessing type I and II fiber size, structure, and distribution of the LMM adjacent to the L5 spinous bilaterally<br>[*Unable to track down authors to see if SD data is available for fiber distribution*]	Mean sizes of type I and II fibers were significantly smaller on the affected side, with a significant difference in mean distribution of type I fibers between sides. A higher frequency of fiber type grouping and small angular fibers was also noted on the side of herniation.<br>**Fiber size** [m ± sd (µm)]:<br>Type I: affected: 58.1 ± 7.5; unaffected: 63.6 ± 8.5 *p<0.01*<br>Type II: affected: 39.3 ± 8.7; unaffected: 44.5 ± 8.5 *p<0.01*<br>**Fiber distribution** [m %]:<br>Type I: affected: 66%; unaffected: 61% *p<0.001*<br>**Frequency of fiber type grouping:**<br>affected: 35%; unaffected: 6% [no statistical analysis]<br>**Frequency of small angular fibers:**<br>affected: 41%; unaffected: 24% [no statistical analysis]

Study	Study population	Comparison / control	Assessment method / variables	Study results
Yoshihara [22]<br><br>[Cross-sectional study]	29 hospital patients with L4/5 LDH and unilateral L5 NR compression<br><br>M/F + 22/7; mean age 36.6 years (21-59 years)<br><br>Mean interval from initial LDH onset to biopsy: 32.2 months (1-240 months)	Patients served as own controls [affected vs. unaffected side analysis]	Biopsy: Histochemical analysis assessing muscle cell type (I and II), size, structure, and groupings of the LMM at L4 and L5, adjacent to the spinous processes bilaterally	In the L5 muscle band, mean sizes of both fiber types on the affected side were sign-ificantly smaller than those on the unaffected side, whereas there was no significant side-to-side difference at L4 and no significant level-to-level difference in the mean sizes of either fiber type on either side (p>0.05).<br><br>Although the percentage of Type I fibers on the side of LDH tended to be higher than on the unaffected side, it was not statistically significant (p>0.05).<br><br>The frequency of small angular fibers and fiber type grouping was not significantly different between sides at L4 (p>0.05), but appeared higher on the side of LDH at L5.<br><br>**Fiber size at L5** [m ± sd (µm)]:<br>Type I: affected: 59.1 ± 7.2; unaffected: 63.1 ± 10.0 *p<0.01*<br>Type II: affected: 40.5 ± 8.1; unaffected: 44.9 ± 7.3 *p<0.001*<br><br>**Frequency of small angular fibers at L5:**<br>Affected: 20.7%; unaffected: 3.4% *[p not provided]*<br><br>**Frequency of fiber type grouping at L5:**<br>Affected: 27.6%; unaffected: 10.3% *[p not provided]*
Zhao [47]<br><br>[Cross-sectional study]	19 hospital patients undergoing surgery for unilateral L4/5 or L5/S1 LDH with evidence of radicular symptoms<br><br>M/F = 13/6; mean age: 43.8 years (21 - 76 years)<br><br>Mean symptom duration: 68.5 months (1 - 240 months)<br><br>9 patients had + SLR on side of LDH	Patients served as own controls [affected vs. unaffected side analysis]	Biopsy: Histochemical analysis assessing proportion of muscle fiber types, size, and strength factor at the transversospinal corner of the LMM bilaterally at LDH level	The % of type I fibers was generally greater on the LDH side, but not significantly so (even after accounting for +/- SLR testing) (p>0.05*)*.<br><br>The CSAs of both fiber types were significantly and consistently smaller on the LDH side, particularly when a + SLR test was present.<br><br>The lesser diameter of both fiber types on the LDH side was significantly smaller than the normal side, but strength factor was only lower in type II fibers on the LDH side.<br><br>**CSA of fiber types** [m ± sd (µm^2)]:<br>Type I: affected: 3315.8 ± 922.7; unaffected: 3757.9 ± 857.2 *p<0.05*<br>Type II: affected: 1931.6 ± 768.9; unaffected: 2321.1 ± 1075.3 *p<0.05*<br><br>**CSA of fiber types (based on SLR findings)** [m ± sd (µm^2)]: *[NB: following values approximated from bar charts]*<br>Type I (+ SLR): affected: 3175 ± 900; unaffected: 3900 ± 950 *p<0.05*<br>Type II (+ SLR): affected: 2000 ± 800; unaffected: 2650 ± 925 *p<0.01*<br><br>**Diameter of fiber types** [m ± sd (µm)]:<br>Type I: affected: 45.3 ± 7.1; unaffected: 48.6 ± 6.8 *p<0.05*<br>Type II: affected: 33.3 ± 7.0; unaffected: 36.5 ± 8.6 *p<0.05*<br><br>**Strength factor** [m ± sd]:<br>Type II: affected: 7.5 ± 3.2; unaffected: 10.0 ± 5.8 *p<0.05*

Assessing muscle changes associated with multiple pathologies via biopsy				
**Study**	**Study population**	**Comparison / control**	**Assessment method / variables**	**Study results**
Jowett [48]<br>[Cross-sectional study]	9 hospital patients undergoing surgery for lumbar spine derangements with positive NR signs<br><br>Mean age: 36.6 years [19 - 58 years]<br><br>Symptom duration and severity not provided	8 patients with same characteristics as primary group, but without positive NR signs.<br><br>Mean age: 33.1 years (15 - 55 years)<br><br>3 cadaveric controls (within 24 hours of death) without lumbar spinal disease	Biopsy: Histochemical analysis assessing fiber count (% slow/fast fibers), cross-sectional fiber area, and slow/fast mean area ratio of the LMM between L2 - L5. No indication of side or relation to which types of underlying pathology were provided	**% of fiber types:**<br>Patients with NR signs: 27% fast fibers<br>Patients without NR signs: 40% fast fibers $p<0.05$<br><br>4 patients with NR signs had whole fascicles consisting of slow fibers only<br><br>**Fiber area:**<br>3 patients with NR signs had a large portion of fast fibers with CSA <1000µ2, as well as being markedly angular.<br><br>*[No data provided or analysis performed]*<br><br>No inferential statistical comparisons were made to the cadaveric group.
	M/F = 13/7; 7 patients with LDH. Other pathologies included: 3 - listhesis; 2 - facet arthrosis; 2 - instability; 1 - lysis; 2 - unspecified. All pathologies were combined, with limited indication as to which were associated with NR signs			

Assessing muscle changes associated with facet arthrosis via imaging

Study	Study population	Comparison / control	Assessment method / variables	Study results
Kalichman [49]<br><br>[Cross-sectional study]	150 medical centre patients with either an abdominal or lumbar CT where assessed for facet arthrosis (≥ grade 2) at L4/5, with < grade 2 serving as the comparison<br>M/F = 82/68; all patients 40+ years of age (mean age (M): 61.74 years; (F): 59.5 years<br><br>Exact number of patients with or without arthrosis was not indicated, but prevalence of arthrosis was ~80% bilaterally and for both sexes		16 or 64 slice unenhanced abdominal or lumbar CT assessing the mean/sd muscle density ratio [RDR] and graded fat infiltration of the LMM and ESM bilaterally at the L4/5 disc level	Statistically significant associations were found between facet arthrosis and the muscle density ratios and grades of fatty infiltration for both muscle groups bilaterally; however, no significant associations were found between arthrosis and mean muscle density on its own.<br><br>**Facet arthrosis association with LMM density (multiple logistic regression analysis)** [odds ratio [95% CI]]:<br><br><u>Density grading:</u><br>(R): 21.13 (3.94-113.38) *p<0.0001*<br>(L): 30.10 (7.37-122.99) *p<0.0001*<br><u>RDR</u>:<br>(R): 0.48 (0.32-0.73) *p=0.001*<br>(L): 0.72 (0.56-0.92) *p=0.009*<br><br>**Facet arthrosis association with ESM density (multiple logistic regression analysis)** [odds ratio [95% CI]]:<br><br><u>Density grading:</u> (R): 38.10 (7.44-199.31) *p<0.0001*; (L): 25.72 (6.47-102.19) *p<0.0001*<br><u>RDR</u>: (R): 0.55 (0.38-0.81) *p=0.002*; (L): 0.66 (0.48-0.91) *p=0.01*
Kalichman [31]<br><br>[cross-sectional data from longitudinal cohort study]	118 general population volunteers with imaging acquired prior to inclusion demonstrating the presence of facet arthrosis (L2/3 - L5/S1)<br><br>Demographics not defined between pathology groupings:<br><br>M/F = 104/83; 40-80 years of age [mean age: 52.6 ± 10.8]; BMI: 27.8± 5.0;<br>37 participants had LBP	69 general population volunteers with imaging acquired prior to inclusion demonstrating the absence of facet arthrosis (L2/3 - L5/S1)	8-slice multi-detector unenhanced abdominal CT measuring the mean density of the LMM and ESM from L3 - L5 [muscle measures matched to side and level of arthrosis]	Statistically significant associations [adjusted for age/sex/BMI] were most consistently found between LMM and ESM density and facet arthrosis at L4/5, particularly with the higher grades. Arthrosis was associated with reduced muscle density.<br><br><u>LMM</u> (L4/5) [mean difference compared to no arthrosis at same side and level]:<br>(R) grade 1 arthrosis: -3.42 *p=0.049*<br>(R) grade 2 arthrosis: -4.43 *p=0.026*<br>(L) grade 3 arthrosis: -7.169 *p=0.0002*<br>(R) grade 3 arthrosis: -4.334 *p=0.029*<br><br><u>ESM</u> [mean difference compared to no arthrosis at same side and level]:<br>L4/5:<br>(L) grade 3 arthrosis: -4.664 *p=0.010*<br>(R) grade 3 arthrosis: -3.529 *p=0.056*<br>L5/S1:<br>(L) grade 1 arthrosis: -4.261 *p=0.023*<br><br>No significant associations were noted for any other outcomes [*p range: 0.079 - 0.983]*.
Kalichman [32]	All details same as above			Both unadjusted simple regression analysis and adjusted multiple regression analysis showed facet arthrosis to be significantly associated with low density LMM and ESM.<br><br>**Unadjusted:** [OR [95% CI]]:<br>LMM: 7.23 [3.05 - 17.10]; ESM: 6.01 [2.64 - 13.70]<br><br>**Adjusted (age, sex, BMI)** [OR [95% CI]]:<br>LMM: 3.68 [1.36 - 9.97]; ESM: 2.80 [1.10 - 7.16]

Study	Study population	Comparison / control	Assessment method / variables	Study results
Sebro [50]<br>[Cross-sectional study]	100 hospital-based patients undergoing CT were assessed for associations of facet arthrosis (L3/4 – L5/S1 combined) on a 0-4 scale, with the lower gradings serving as the comparison. No specific breakdown of the number of patients with various arthrosis scores was provided<br><br>M/F = 51/49; mean age: 44.4 ± 22.2 years; mean BMI: 27.8 ± 7.4<br><br>Symptom duration and severity not reported		Abdominal or pelvic axial CT assessing the muscle density of the LMM, PMM and longissimus bilaterally at L3/4 & L4/5 combined	Statistically significant negative correlations were found between paraspinal muscle density and facet arthrosis (with univariate regression analysis); however, paraspinal muscle density was not a significant predictor of facet arthrosis with multivariate analysis.<br><br>**Univariate analysis** [correlation values]:<br>PMM: -0.59 *p<0.0001*<br>LMM: -0.61 *p< 0.0001*<br>Longissimus: -0.68 *p<0.0001*<br><br>**Multivariate analysis** [p values only]:<br>PMM: *p=0.72*<br>LMM: *p=0.89*<br>Longissimus: *p=0.86*
Yu [51]<br>[Cross-sectional study]	160 hospital-based patients (Jan. 2008 - Dec. 2014) with acute or chronic LBP (and MRIs and CTs of the lumbar region) were assessed for facet arthrosis from L3/4 – L5/S1 (from CT) on a 0-3 scale, with 0-1 gradings serving as the comparison [the distribution of patients with arthrosis per level was: L3/4 – 31.9%; L4/5 – 53.1%; L5/S1 – 49.4%]<br><br>M/F = 74/86; mean age: 47.3 ± 12.7 years; mean BMI: 20.9 ± 3.2<br><br>Patient distribution (based on reason for MRI): acute LBP: 25; chronic LBP: 96; exclude fracture: 20; other: 19 [symptom duration not reported]		MRI T2W FSE axial images assessing CSA (TCSA:VCSA ratio), muscle-fat index (MFI), CSA asymmetry and muscle-fat index asymmetry of the LMM bilaterally at the superior endplate levels of L4, L5, & S1; multi-slice abdominal CT for grading facet arthrosis [muscle measures matched to side and level of arthrosis]	The presence of arthrosis resulted in significantly smaller CSA and significantly higher MFI at all levels compared to the absence of arthrosis. Significantly greater CSA asymmetry was noted at L5/S1 and higher MFI asymmetry was noted at L4/5 on the right, when arthrosis was present.<br><br>**CSA**: [m ± sd]<br>L3/4: No OA / OA (L): 0.35±0.07 / 0.29±0.08; (R): 0.36±0.08 / 0.29±0.07<br>L4/5: No OA / OA (L): 0.52±0.11 / 0.41±0.10; (R): 0.52±0.11 / 0.42±0.10<br>L5/S1: No OA / OA (L): 0.61±0.11 / 0.51±0.11; (R): 0.61±0.11 / 0.49±0.12 [*all p values <0.001*]<br><br>**CSA asymmetry**: [m (%) ± sd (%)]<br>L5/S1: No OA / OA (L): 5.5 ± 5.1 / 8.6 ± 6.3 *p=0.002;* (R): 5.4 ± 5.0 / 8.8 ± 6.2 *p=0.001*<br><br>**MFI**: [m ± sd]<br>L3/4: No OA / OA (L): 0.07±0.03 / 0.14±0.06; (R): 0.07±0.04 / 0.15±0.06<br>L4/5: No OA / OA (L): 0.08±0.03 / 0.15±0.07; (R): 0.08±0.04 / 0.15±0.06<br>L5/S1: No OA / OA (L): 0.10±0.04 / 0.18±0.08; (R): 0.09±0.03 / 0.18±0.07 [*all p values <0.001*]<br><br>**MFI asymmetry**: [m (%) ± sd (%)]<br>L4/5: No OA / OA (L): 14.5 ± 11.0 / 12.5 ± 9.1 *p=0.05;* (R): 14.3 ± 12.1 / 11.9 ± 9.2 *p=0.04*<br><br>Smaller CSA at L4/5 and greater CSA asymmetry at L5/S1 were independently associated with arthrosis. A higher MFI was independently associated with arthrosis at all 3 spinal levels.<br><br>**CSA**:<br>**Univariate analysis** [Odds ratio (95%CI): L4/5: 0.1 (0.1-0.3) *p<0.001*<br>**Multivariate analysis** [Odds ratio (95%CI)]: L4/5: 0.2 (0.1-0.6) *p=0.005*<br><br>**CSA asymmetry**:<br>**Univariate analysis** [Odds ratio (95%CI): L5/S1: 3.7 (1.8-7.5) *p<0.001*<br>**Multivariate analysis** [Odds ratio (95%CI)]: L5/S1: 3.9 (1.4-10.6) *p=0.009*<br><br>**MFI**:<br>**Univariate analysis** [Odds ratio (95%CI):<br>L3/4: 11.6 (4.7-28.7); L4/5: 11.1 (4.9-25.1); L5/S1: 14.9 (6.0-36.8) [all *p<0.001*]<br>**Multivariate analysis** [Odds ratio (95%CI)]:<br>L3/4: 15.8 (3.2-77.8) *p=0.001*; L4/5: 6.1 (1.7-21.6)) *p=0.005*; L5/S1: 8.5 (2.9- 25.3) *p<0.001*

**Assessing muscle changes associated with canal stenosis via imaging**				
**Study**	**Study population**	**Comparison / control**	**Assessment method / variables**	**Study results**
Abbas [52]<br><br>[Cross-sectional study]	165 hospital patients with symptoms of spinal stenosis<br><br>M/F = 80/85; age range: 40-88 years; mean BMI: ~30.5<br><br>Symptom duration and severity not reported	180 hospital patients without spinal stenosis or LBP symptoms<br><br>M/F = 90/90; age range: 40-99 years; mean BMI: ~27.5	Non-contrast axial CT assessing FCSA and fat infiltration of the PMM, LMM, ESM at L3 bilaterally	**Density**: The stenosis group had significantly higher muscle density compared to the control group (adjusted for age and BMI).<br><br>Control / Stenosis [m ± sd (HU)]:<br>PMM (M): 40 ± 9 / 45 ± 9 **; (F): 40 ± 8 / 43 ± 10 *<br>LMM (M): 34 ± 15 / 45 ± 12 **; (F): 24 ± 16 / 31 ± 17 **<br>ESM (M): 34 ± 11 / 43 ± 10 **; (F): 29 ± 12 / 35 ± 15 ** **p = 0.01; ** p <0.001*<br><br>LMM densities for Male and Female, and ESM density for males increased the likelihood of symptomatic stenosis development [OR (95% CI)].<br><br>LMM (M): 1.12 (1.023–1.165); *p=0.007;* (F): 1.10 (1.032–1.120); *p<0.001*<br>ESM (M): 1.12 (1.004–1.177); *p=0.039*<br><br>**FCSA:** Values were significantly greater in the Male stenosis group for ESM & PMM, and the Female stenosis for ESM; however, no significant difference between groups was noted for the LMM (p range = 0.163 – 0.331).<br><br>Control / Stenosis [m ± sd (mm^2)]:<br>PMM (M): 1026 ± 276 / 1097 ± 234; *p=0.042;* (F): 628 ± 168 / 698 ± 145; *p=0.076*<br>ESM (M): 1662 ± 394 / 1793 ± 369; *p=0.011;* (F): 1345 ± 338 / 1540 ± 314; *p=0.014*
Jiang [53]<br><br>[Cross-sectional study]	40 pre-surgical hospital patients with clinical and MRI evidence of spinal stenosis and neural compression at L4/5<br><br>M/F = 16/24; mean age:61.5 ± 3.4 years; mean BMI: 23.3 ± 1.7<br><br>Symptom duration and severity not reported	40 patients undergoing spinal MRI without LBP or neurological symptoms<br><br>M/F = 18/22; mean age: 60.4 ± 3.3 years; mean BMI: 22.9 ± 1.6	T2W and T1W MRI assessing TCSA, TCSA/VB ratio, fat infiltration ratio, and asymmetry of the LMM at L4/5 bilaterally	**Fat infiltration**: The fat infiltration ratio was significantly greater in the stenosis group.<br>Control / Stenosis (m ± sd):<br>0.219 ± 0.052 / 0.264 ± 0.069; *p=0.0043*<br><br>**Asymmetry**: muscle asymmetry was significantly greater in the stenosis group.<br>Control / Stenosis (m ± sd):<br>0.031 ± 0.021 / 0.049 ± 0.032; *p=0.0057*<br><br>**Muscle size**: The TCSA was not significantly different between groups; however, the TCSA/VB ratio was significantly lower in the stenosis group.<br><br><u>TCSA</u>:<br>Control / Stenosis [m ± sd (cm^2)]:<br>16.685 ± 1.886 / 15.850 ± 2.286; *p=0.0876*<br><br><u>Ratio</u>:<br>Control / Stenosis [m ± sd]:<br>0.866 ± 0.092 / 0.807 ± 0.094; *p=0.0064*

Study	Study population	Comparison / control	Assessment method / variables	Study results
Kalichman [31]<br><br>[cross-sectional data from longitudinal cohort study]	15 general population volunteers with imaging acquired prior to inclusion demonstrating the presence of spinal stenosis)<br><br>Demographics not defined between pathology groupings:<br><br>M/F = 104/83; 40-80 years of age [mean age: 52.6 ± 10.8]; BMI: 27.8± 5.0<br>37 participants had LBP	172 volunteers with imaging acquired prior to inclusion demonstrating the absence of spinal stenosis	8-slice multi-detector unenhanced abdominal CT measuring the mean density of the LMM and ESM from L3 - L5	No statistically significant associations with muscle density were found with spinal stenosis at any level for either muscle type, after adjusting for age, sex, and BMI.<br><br>**Adjusted LMM density associations with spinal stenosis** [estimate (*p value*)]:<br>L3: 2.816 *p=0.443*<br>L4: -0.515 *p=0.899*<br>L5: 0.316 *p=0.931*<br><br>**Adjusted ESM density associations with spinal stenosis** [estimate (*p value*)]:<br>L3: -0.090 *p=0.971*<br>L4: -2.721 *p*=0.424<br>L5: -1.515 *p=0.721*
Kalichman [32]	All details same as above			Adjusted multiple regression analysis showed that stenosis was not significantly associated with lower density of the LMM and ESM.<br><br>**Spinal stenosis association with muscle density (adjusted)** [odds ratio [95% CI]]:<br>LMM: 2.15 [0.54 - 8.63]<br>ES: 1.73 [0.46 - 6.55
Ogon [54]<br><br>[Cross-sectional study]	40 patients with lumbar spinal stenosis and VAS score <30 [stenosis defined clinically; not specific to any level]<br><br>M/F = 17/23; mean age: 65.0 ± 1.2 years [range: 42-77]; BMI: 23.3 ± 0.5<br><br>VAS: 12.3 ± 1.3	40 patients with non-specific, chronic LBP and VAS score >30<br><br>M/F = 16/24; mean age: 62.9 ± 1.9 years [range: 41-79]; BMI: 23.9 ± 0.6<br><br>VAS: 68.1 ± 2.6	MRI spectroscopy assessing mean intramyocellular lipid (IMCL) vs extramyocellular lipid (EMCL) content of the LMM at L4/5 on the right	The mean IMCL content was significantly higher in the CLBP group, but the mean EMCL content was not significantly different between groups.<br><br>**IMCL**: [m ± se of mean] [($x10^2$) mmol/l]<br>CLBP: 11.6 ± 1.85<br>Stenosis: 4.70 ± 1.13 *p<0.01*<br><br>**EMCL**: [m ± se of mean] [($x10^3$) mmol/l]<br>CLBP: 4.72 ± 0.57<br>Stenosis: 4.77 ± 0.50 *p=0.71*
Yarjanian [20]<br><br>[Cross-sectional study]	15 university hospital patients with clinical and MRI confirmed stenosis at any spinal level<br><br>M/F = 5/10; mean age: 68.53 ± 8.54 years; mean VAS: 4.41 ± 3.16<br><br>Symptom duration not reported	10 university hospital patients with non-radiating LBP but no spinal stenosis on MRI<br>M/F = 4/6; mean age: 62.00 ± 8.58 years; mean VAS: 3.28 ± 2.22<br>———<br>10 asymptomatic volunteers from local community<br>M/F = 5/5; mean age: 65.00 ± 6.72 years; VAS: 0	MRI T2 axial images assessing FCSA of the LMM bilaterally at L5/S1 level (to include aspects of L3-L5 muscles); used combined bilateral and combined assessor measures	A significant difference was found in the FCSA between the control and stenosis groups only.<br><br>**FCSA per group** [m ± sd [mm^2]]:<br>Control (C): 3872.80 ± 892.84<br>LBP (L): 3627.60 ± 990.38<br>Stenosis (S): 2985.80 ± 760.78<br>Differences between groups:<br>C:L (*p =0.80); * C:S (*p =0.04);* L:S (*p =0.18)*<br><br>Correlations between the smallest AP canal diameter and FCSA were weak and insignificant [R=0.08; *p=0.76*].<br><br>The FCSA reduced as the severity of stenosis increased, but not significantly [ANOVA: *p=0.66*].

LMM: Lumbar multifidus muscle; PMM: Psoas major muscle; PVM: Paravertebral (paraspinal) muscle; ESM: Erector spinae muscle; QLM: Quadratus lumborum muscle; (C)LBP: (Chronic) low back pain; NR: Nerve root; OA: Osteoarthrosis (osteoarthritis), including facet arthrosis; HNP/LDH: Herniated nucleus pulposus / lumbar disc herniation; VAS: Visual analogue scale; ODI: Oswestry disability index; SLR: Straight leg raise; C: Control / comparison group; CSA: Cross-sectional area; TCSA: Total CSA; FCSA: Functional CSA; VCSA: Vertebral body CSA; MLD: Muscle laminar distance [for consistency, all various classifications of total area or muscle-only CSA measures are referred to by the abbreviations: TCSA and FCSA, respectively].

† Where studies have assessed pathologies and/or muscles outside the scope of this review, only those components relative to this review were included.
